# Persister cell phenotypes contribute to poor patient outcomes after neoadjuvant chemotherapy in PDAC

**DOI:** 10.1038/s43018-023-00628-6

**Published:** 2023-09-07

**Authors:** Xu Zhou, Jingyu An, Roma Kurilov, Benedikt Brors, Kai Hu, Teresa Peccerella, Stephanie Roessler, Katrin Pfütze, Angela Schulz, Stephan Wolf, Nicolas Hohmann, Dirk Theile, Max Sauter, Jürgen Burhenne, Shigenori Ei, Ulrike Heger, Oliver Strobel, Simon T. Barry, Christoph Springfeld, Christine Tjaden, Frank Bergmann, Markus Büchler, Thilo Hackert, Franco Fortunato, John P. Neoptolemos, Peter Bailey

**Affiliations:** 1 Department of General, Visceral and Transplantation Surgery, Heidelberg University Hospital, Heidelberg, Germany; 2 Section Surgical Research, University Clinic Heidelberg, Heidelberg, Germany; 3 Division of Applied Bioinformatics, The German Cancer Research Center (DKFZ), Heidelberg, Germany; 4 German Cancer Consortium (DKTK), Core Center Heidelberg, Heidelberg, Germany; 5 National Center for Tumour Disease (NCT), Heidelberg, Germany; 6 Institute of Pathology, Heidelberg University Hospital, Heidelberg, Germany; 7 Department of Translational Medical Oncology, National Center for Tumor Diseases, Heidelberg University Hospital, The German Cancer Research Center (DKFZ), Heidelberg, Germany; 8 NGS Core Facility, The German Cancer Research Center (DKFZ), Heidelberg, Germany; 9 Department of Medical Oncology, National Center for Tumor Diseases, Heidelberg University Hospital, Heidelberg, Germany; 10 Department of Clinical Pharmacology and Pharmacoepidemiology, Heidelberg University Hospital, Heidelberg, Germany; 11 Department of Gastroenterological Surgery, Tokai University School of Medicine, Kanagawa, Japan; 12 Department of General Surgery, Division of Visceral Surgery, Medical University of Vienna, Vienna, Austria; 13 Bioscience, Early Oncology, AstraZeneca, Cambridge, UK; 14 Botton-Champalimaud Pancreatic Cancer Center, Lisbon, Portugal; 15 Department of General, Visceral and Thoracic Surgery, Medical Center Hamburg-Eppendorf, Hamburg, Germany; 16 School of Cancer Sciences, University of Glasgow, Glasgow, UK

**Keywords:** Pancreatic cancer, Tumour biomarkers, Cancer

## Abstract

Neoadjuvant chemotherapy can improve the survival of individuals with borderline and unresectable pancreatic ductal adenocarcinoma; however, heterogeneous responses to chemotherapy remain a significant clinical challenge. Here, we performed RNA sequencing (*n* = 97) and multiplexed immunofluorescence (*n* = 122) on chemo-naive and postchemotherapy (post-CTX) resected patient samples (chemoradiotherapy excluded) to define the impact of neoadjuvant chemotherapy. Transcriptome analysis combined with high-resolution mapping of whole-tissue sections identified GATA6 (classical), KRT17 (basal-like) and cytochrome P450 3A (CYP3A) coexpressing cells that were preferentially enriched in post-CTX resected samples. The persistence of GATA6^hi^ and KRT17^hi^ cells post-CTX was significantly associated with poor survival after mFOLFIRINOX (mFFX), but not gemcitabine (GEM), treatment. Analysis of organoid models derived from chemo-naive and post-CTX samples demonstrated that CYP3A expression is a predictor of chemotherapy response and that CYP3A-expressing drug detoxification pathways can metabolize the prodrug irinotecan, a constituent of mFFX. These findings identify CYP3A-expressing drug-tolerant cell phenotypes in residual disease that may ultimately inform adjuvant treatment selection.

## Abstract (web summary)

Zhou et al. investigate the molecular events that follow neoadjuvant treatment in PDAC patients, explore signatures of heterogeneous response and persister phenotypes, identify a CYP3A link to resistance and suggest that it is useful in predicting response.

## Main

Pancreatic ductal adenocarcinoma (PDAC) is an increasing oncological challenge that requires a deeper understanding of its resistance to treatment. For all stages combined, the 5-year survival rate is only 11% for pancreatic cancer in the United States, which is the third most frequent cause of death from cancer (https://gco.iarc.fr/overtime)^1^. Despite modest improvements in survival due to treatments based on systemic chemotherapy, most individuals with metastatic pancreatic cancer will still die within 12 months, with no long-term survivors^2–4^. The best progress has been made in individuals with locally resectable tumors, which is attributable to improvements in surgical techniques and the use of adjuvant systematic chemotherapy^5–9^. Despite having increased estimated 5-year survival rates from 8% with resection alone to 30–50% in conjunction with adjuvant chemotherapy, most individuals relapse within a median of 12.8–21.6 months (refs. ^5,8,9^).

Individuals with borderline resectable disease seem to have a better survival benefit from neoadjuvant gemcitabine (GEM) with capecitabine or mFOLFIRINOX (mFFX) rather than with chemoradiotherapy^10–12^. Induction therapy may also increase resectability and improve survival in individuals with otherwise unresectable local disease^9,13,14^. Although second-line cytotoxic therapies are also delivered after disease progression or relapse in metastatic, locally advanced and postresection settings, response to treatment and overall survival are disappointing compared to other tumor types^5,8,15^.

Dissociated responses are observed in individuals with metastatic disease, in which some metastases respond to treatment whereas others remain stable or progress. Chemoradiotherapy may also be implicated in the differential prognosis of pathological treatment effects in individuals who have received neoadjuvant chemotherapy^14^. Single-nucleus and spatial transcriptomic profiling of chemo-naive and post-therapy (chemoradiotherapy and chemotherapy) samples has identified distinct neoplastic cell phenotypes that exist in untreated samples and persist after therapy^16^. Systematic profiling of metastatic biopsies and matched organoid models has identified a continuum of transcriptional states spanning classical and basal-like phenotypes that are joined by an intermediate coexpressor (IC) or ‘hybrid’ transcriptional state^17^. Ex vivo studies have further revealed that transitions between classical and basal-like phenotypes may significantly impact drug responses, with basal-like states exhibiting broadly decreased sensitivity to chemotherapy^17^.

Despite these advances, the contribution of distinct neoplastic cell phenotypes to outcomes following neoadjuvant chemotherapy remains largely underexplored. Here, we identify GATA6-, KRT17- and cytochrome P450 3A (CYP3A)-coexpressing cells that are enriched in resected PDAC samples following neoadjuvant chemotherapy. We also reveal a tumor cell-intrinsic role for CYP3A-expressing detoxification pathways in the persistence of drug-tolerant cells in minimal residual disease.

## Results

### The PDAC Heidelberg (PDAC-HD) sample cohort

**Fig. 1:**
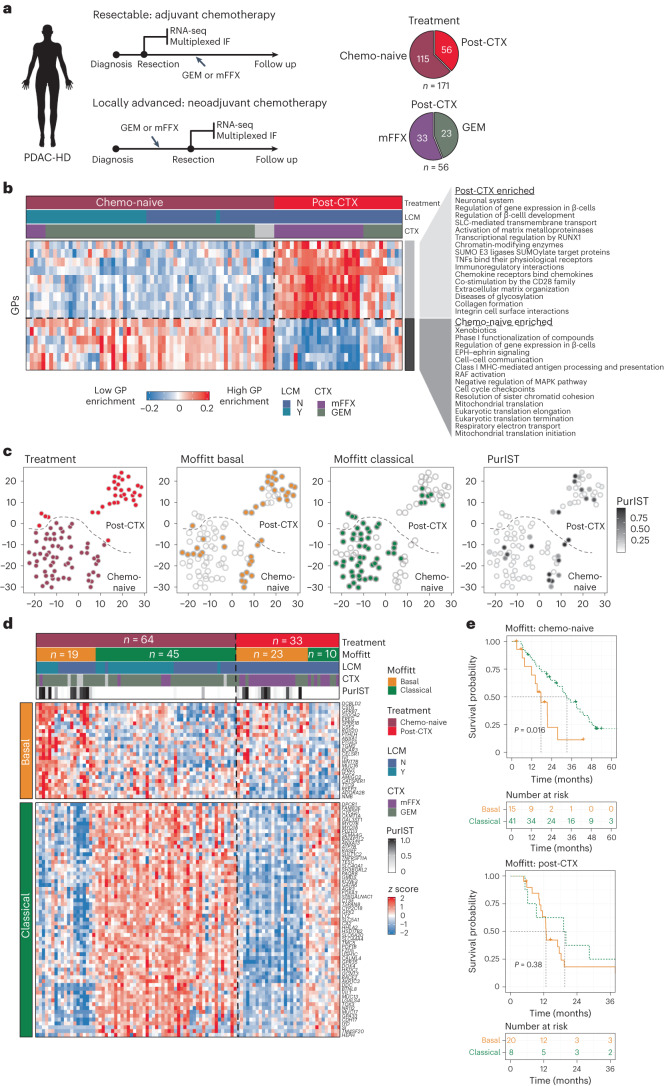
Transcriptomic profiling of chemo-naive and post-CTX PDAC-HD samples. **a**, The PDAC-HD cohort included 171 unique samples representing 115 chemo-naive and 56 post-CTX resections. Post-CTX samples received either GEM (*n* = 23) or mFFX (*n* = 33). These samples were analyzed by RNA-seq or multiplexed IF. The RNA-seq set was composed of a total of *n* = 97 samples, including *n* = 64 chemo-naive and *n* = 33 post-CTX samples. LCM was performed on chemo-naive samples (*n* = 32). Chemo-naive individuals received adjuvant GEM (*n* = 54) and mFFX (*n* = 5). Post-CTX individuals received neoadjuvant GEM (*n* = 10) and mFFX (*n* = 23). **b**, WGCNA of RNA-seq data showing significantly enriched GPs between chemo-naive and post-CTX PDAC-HD samples. Significance was determined by two-sided Wilcoxon rank-sum test adjusted for multiple testing (*P* ≤ 0.05). The heat map shows relative module eigengene expression between chemo-naive and post-CTX samples, with red (positive) values associated with increased GP expression and blue (negative) values associated with decreased GP expression. Molecular function and biological processes associated with GPs and enriched in chemo-naive or post-CTX samples are shown; Y, yes; N, no; TNF, tumor necrosis factor; MHC, major histocompatibility complex. **c**, *t*-distributed stochastic neighbor embedding (t-SNE) plots showing samples clustered according to the 2,000 top variably expressed genes. Sample clustering is identical between plots, with Moffitt classification and PurIST scores indicated for each sample. **d**, Heat map showing the classification of chemo-naive and post-CTX PDAC-HD samples by Moffitt subtype. **e**, Kaplan–Meier survival analysis of chemo-naive and post-CTX PDAC-HD samples. The log-rank *P* values are annotated on the plots. *P* values were not adjusted for multiple testing. Source data

To define the impact of neoadjuvant chemotherapy, we established a PDAC-HD cohort with validated PDAC tissue and defined pathological clinical characteristics, including long-term follow-up in 171 individuals comprising (1) chemo-naive PDAC tissue after primary resection in individuals with radiologically resectable tumors with most receiving adjuvant chemotherapy (*n* = 115) after resection and (2) postchemotherapy (post-CTX) PDAC tissue after resection following neoadjuvant therapy in individuals with radiologically borderline resectable or unresectable tumors (*n* = 56 (refs. ^13,18^); Fig. 1a). Individuals who received chemoradiotherapy were excluded from the study.

**Extended Data Fig. 1:**
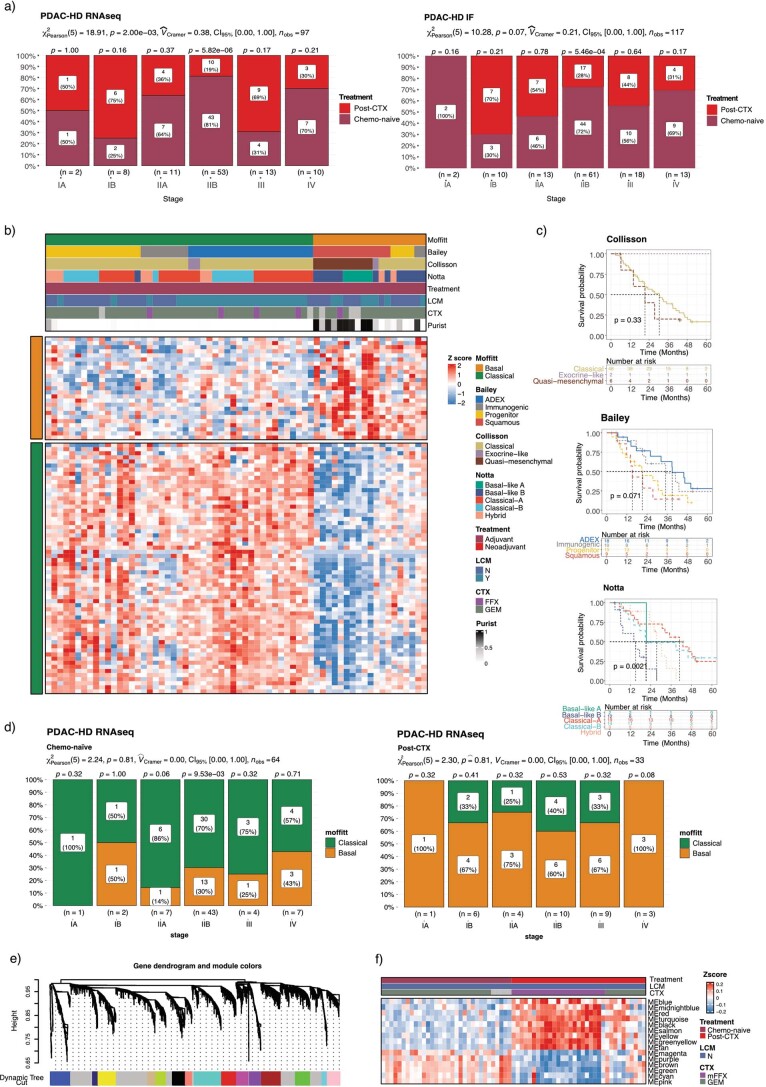
Subtype and transcriptomic analysis of PDAC-HD samples. **a**) Bar stat plots showing the number of chemo-naïve and post-CTX samples belonging to a defined AJCC (8^th^ Edition) Stage^19^ in either the PDAC-HD RNAseq or PDAC-HD IF sample cohorts. Stage (I-IV) and corresponding sample number (n) are shown at the bottom of each plot. A Pearson χ^2^-test of independence (two-sided) is provided at the top of each plot and *P*-values from a one sample proportions test are displayed on the top of each bar (two-sided). *P*-values are not adjusted for multiple testing. **b**) Heatmap showing Moffitt classification of chemo-naïve PDAC-HD samples using established subtyping schemes. The heatmap is annotated with Bailey, Collisson and Notta subtyping designations and PurIST scores. **c**) Kaplan Meier survival analysis of chemo-naive PDAC-HD samples stratified by the Bailey, Collisson and Notta subtyping schemes. The number of patients falling into one of the designated subtypes is shown in the ‘Number at risk’ table. Log-rank test (two-sided) *P*-values are provided for each comparison. Log-rank *P*-values are not adjusted for multiple correction. **d**) Bar stat plots showing the number of patient samples belonging to a Moffitt subtype and grouped by AJCC Stage. Stage (I-IV) and corresponding sample number (n) are shown at the bottom of each plot. Separate plots are provided for chemo-naïve and post-CTX samples. As above, a Pearson χ^2^-test of independence (two-sided) is provided at the top of each plot and *P*-values from a one sample proportions test (two-sided) are displayed on the top of each bar. *P*-values are not adjusted for multiple testing. **e**) WGCNA dendrogram of co-expressed genes showing dissimilarity based on topological overlap and assigned gene module colours. **f**) Heatmap showing the enrichment of gene programs in chemo-naïve and post-CTX patient samples. Samples are clustered by module eigengene values with higher values (red) associated with increased enrichment and lower values (blue) associated with decreased enrichment of a gene module in a sample. The gene modules presented are all significantly enriched. LCM patient samples were removed from the analysis to provide a direct comparison of chemo-naïve and post-CTX bulk RNAseq samples. Source data

RNA sequencing (RNA-seq) was performed on cryopreserved PDAC tissues from 97 individuals, and multiplexed immunofluorescence (IF) was performed on PDAC formalin-fixed paraffin-embedded (FFPE) tissues from 122 individuals and on normal pancreas tissues from 9 organ donors. RNA-seq and multiplex IF were performed on identical samples from 48 individuals. Laser-capture microdissection (LCM) was performed on 32 chemo-naive samples, which were analyzed by RNA-seq. Assessment of clinical stage^19^ demonstrated that chemo-naive and post-CTX individuals had similar numbers of late-stage tumors (stage III and IV), although a higher number of stage IIB tumors were present in the chemo-naive group (Extended Data Fig. 1a). Chemo-naive samples selected for RNA-seq and IF analysis received GEM monoadjuvant chemotherapy almost exclusively, with a small number of individuals receiving either mFFX (RNA-seq: 5 of 60; IF: 11 of 76) or GEM nab-paclitaxel (RNA-seq: 3 of 60; IF: 5 of 76). Post-CTX samples received either mFFX or GEM nab-paclitaxel. Most individuals who received adjuvant postoperative chemotherapy were placed on the original neoadjuvant chemotherapy regimen. Demographic characteristics are presented in Supplementary Tables 1 and 4.

Post-CTX samples were also retrospectively reviewed for College of American Pathologists (CAP) scoring for histological treatment effect in response to neoadjuvant chemotherapy as a prognostic factor for individuals undergoing surgical resection for localized PDAC^14^. CAP scoring was performed by a specialist pancreatic cancer pathologist (F.B.). Only one individual out of a total of 32 evaluable participants receiving neoadjuvant chemotherapy exhibited a near-complete response (score 1), 19 exhibited a partial response (score 2), and the 12 remaining individuals exhibited no response (score 3; Supplementary Table 4).

### RNA-seq profiling of chemo-naive and post-CTX PDAC-HD samples

RNA-seq was initially performed to define the neoplastic and stromal changes associated with neoadjuvant chemotherapy (Supplementary Table 6). Weighted gene coexpression analysis (WGCNA) of bulk RNA-seq data demonstrated that neoadjuvant chemotherapy profoundly impacted the composition of both tumor stroma and neoplastic cell populations (Fig. 1, Extended Data Fig. 1e,f and Supplementary Tables 7 and 8). Importantly, the removal of LCM-derived samples from the analysis did not change the significance of the results, suggesting that sample preparation was not a confounding factor in this study (Supplementary Table 8).

WGCNA identified 15 coordinately expressed gene programs (GPs) representing distinct biological processes that could discriminate chemo-naive and post-CTX samples (Fig. 1b, Extended Data Fig. 1e,f and Supplementary Table 8). Nine core GPs encompassing the regulation of β-cell development, extracellular matrix organization, collagen formation, immunoregulatory interactions and chemokine signaling were significantly enriched in the post-CTX samples. By contrast, six GPs encompassing cell cycle checkpoints, resolution of sister chromatid cohesion, xenobiotics and phase I functionalization of compounds, cell–cell communication and Eph–ephrin, RAF and MAPK signaling pathways were significantly repressed in response to neoadjuvant chemotherapy.

**Extended Data Fig. 2:**
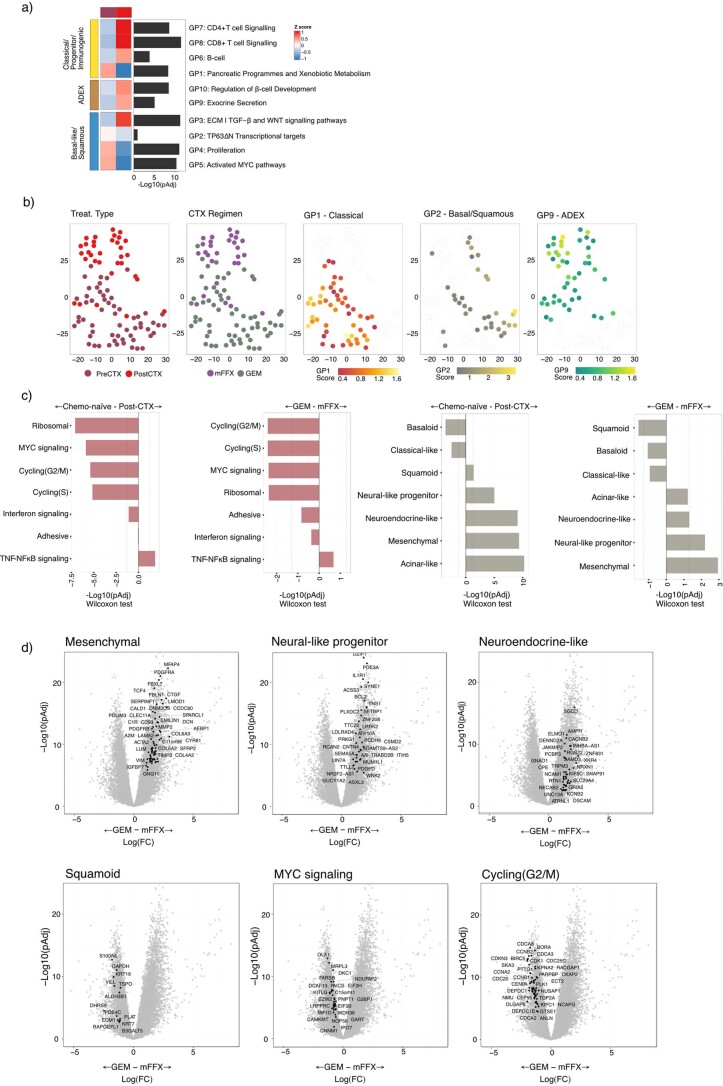
Enrichment of subtype-specific gene programs (GP) and neoplastic cell phenotypes in chemo-naïve and post-CTX samples. **a)** Heatmap and bar plot of previously defined Bailey GP signatures that are significantly enriched in either the Classical/Progenitor/Immunogenic, Basal-like/Squamous or ADEX subtypes. Heatmap z scores represent changes in median GP scores between chemo-naïve (n = 64) and post-CTX (n = 33) samples. Bar plots represent –Log_10_ (Kruskal Wallis rank sum test two-sided *P*-value adjusted for multiple testing (pAdj)). **b**) Single sample gene set enrichment analysis using GP signatures (as shown in a) followed by tSNE. Sample clusters are identical between tSNE plots with individual samples highlighted according to treatment type, chemotherapy regimen and indicated GP score. Analyses for chemo-naïve (n = 64) and post-CTX (n = 33) samples are shown. **c**) Bar charts showing the differential enrichment of specific neoplastic cell populations in chemo-naïve and post-CTX samples as indicated. The significance of the enrichment is provided as -Log10(Wilcoxon rank sum test two-sided pAdj-value adjusted for multiple testing). The dotted line represents -Log10(pAdj ≤ 0.05). **d**) Volcano plots showing the differential enrichment of genes associated with the indicated neoplastic cell populations in post-CTX samples treated with either GEM or mFFX. Genes significantly enriched (Log 2 Fold change > 1 and -Log_10_(pAdj) >2) in samples treated pre-operatively with GEM or mFFX are shown. pAdj represent the significance of a two-sided Wald test adjusted for multiple testing. Source data

Enrichment analysis using gene signatures for established PDAC subtypes demonstrated that classical, basal-like and ADEX (aberrantly differentiated endocrine exocrine) GPs persisted in selected post-CTX samples (Fig. 1c and Extended Data Fig. 2a,b). Extending this analysis to recently defined single-cell phenotypes^16^ revealed a significant enrichment of gene signatures encompassing ADEX-like (neuroendocrine-like and acinar-like), squamous (mesenchymal and squamoid) and neural-like progenitor cell phenotypes in post-CTX resected samples (Extended Data Fig. 2c,d). Basaloid and classical-like cell gene signatures were significantly enriched in chemo-naive samples but were depleted in post-CTX samples. A comparative analysis of post-CTX GEM and mFFX samples demonstrated that neural-like progenitor, mesenchymal and neuroendocrine-like cell types were enriched in samples treated preoperatively with mFFX, while the squamoid cell type was significantly enriched in GEM post-CTX samples (Extended Data Fig. 2c,d). Analysis of neoplastic cell state signatures^16^ demonstrated that cycling (G2/M), cycling (S), MYC signaling and ribosomal cell states were all significantly enriched in GEM post-CTX samples (Extended Data Fig. 2c,d). These findings suggest that different chemotherapies may enrich for distinct neoplastic cell phenotypes.

**Fig. 2:**
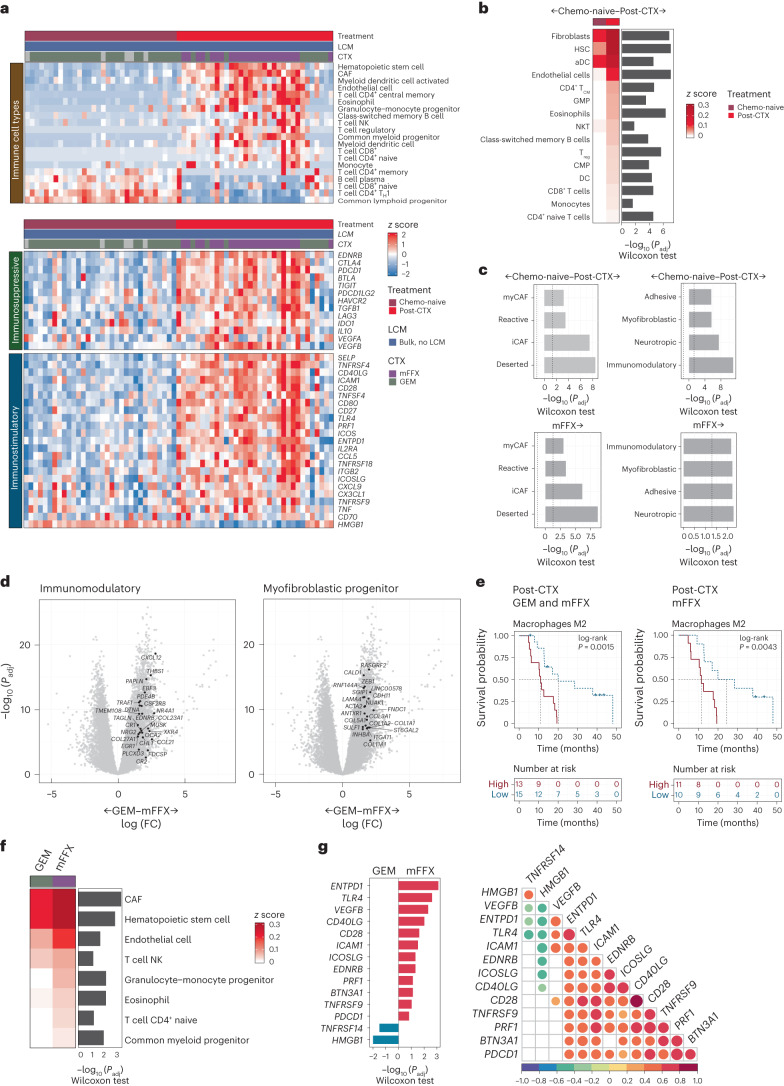
Neoadjuvant chemotherapy impacts the TME. **a**, Top, significantly enriched immune cell types. Middle, significantly expressed immunosuppressive genes. Bottom, significantly expressed immunostimulatory genes. Significance was determined by two-sided Wilcoxon rank-sum test adjusted for multiple testing (*P* ≤ 0.05); T_H_1, type 1 helper T cell. **b**, Immune cell types that exhibit significant enrichment in chemo-naive (*n* = 32) and post-CTX (*n* = 33) samples. The heat map represents median immune cell enrichment, and the bar chart represents significance of enrichment as –log_10_ (Wilcoxon rank-sum test two-sided *P* value adjusted for multiple testing (*P*_adj_)); HSC, hematopoeitc stem cell; aDC, activated dendritic cell; T_CM_, central memory T cell; GMP, granulocyte–monocyte progenitor; T_reg_, regulatory T cell; CMP, common myeloid progenitor. **c**, Bar charts showing significant enrichment of specific stromal signatures in post-CTX samples; myCAFs, myofibroblast-like CAFs; iCAFs, inflammatory CAFs. The significance is provided as –log_10_ (Wilcoxon rank-sum test two-sided *P* value adjusted for multiple testing). The dotted line represents –log_10_ (*P*_adj_ ≤ 0.05). **d**, Volcano plots showing the enrichment of immunomodulatory and myofibroblastic cell signature genes in samples treated preoperatively with GEM or mFFX. Genes significantly enriched (log_2_ (fold change) of >1 and –log_10_ (*P*_adj_) of >2) in post-CTX mFFX samples are shown. *P*_adj_ represent the significance of a two-sided Wald test adjusted for multiple testing; FC, fold change. **e**, Kaplan–Meier survival analysis for high (greater than median) and low (less than median) macrophage M2 enrichment values in post-CTX (GEM and mFFX) and mFFX PDAC-HD samples. Participant numbers for each group are provided under ‘Numbers at risk’. A log-rank (two-sided) *P* value of ≤0.05 is considered significant. **f**, Immune cell types that exhibit significant enrichment in mFFX post-CTX samples (*n* = 23) relative to GEM post-treated samples (*n* = 10). The heat map represents median immune cell enrichment, and the bar chart represents the significance of enrichment as –log_10_ (Wilcoxon rank-sum test two-sided *P* value adjusted for multiple testing). **g**, Immunostimulatory genes that are significantly and differentially expressed between post-CTX GEM (*n* = 10) and mFFX (*n* = 23) samples. The bar chart provides the significance of enrichment as –log_10_ (Wilcoxon rank-sum test two-sided *P* value adjusted for multiple testing). Correlation heat map showing correlations between immunomodulatory factors in post-CTX samples. Pearson’s correlations are shown in the plot. Significance was determined by two-sided Pearson’s correlation test. *P* values were not adjusted for multiple testing. All correlations shown are significant. Source data

Marked changes in cellular composition and tumor–stroma interactions have been previously observed in post-CTX resected samples^20–23^. Stromal GPs that were significantly enriched post-CTX included those commonly associated with wound healing and fibrosis (that is, extracellular matrix deposition and inflammation; Fig. 1b and Supplementary Table 8). Cancer-associated fibroblasts (CAFs), endothelial cells and several immune cell subsets were significantly enriched post-CTX (Fig. 2a,b). A comparison of neoadjuvant chemotherapy regimens revealed that mFFX ‘educated’ the tumor microenvironment (TME) in a markedly different way than GEM (Fig. 2c,d,f,g). Preoperative treatment with mFFX preferentially enriched for CAF signatures^16^ (representing immunomodulatory, myofibroblastic progenitor, adhesive and neurotropic fibroblast programs), immune signatures (representing common myeloid progenitors, granulocyte–monocyte progenitors, T cell natural killer (NK) and T cell CD4^+^ naive) and immunomodulatory factors, including VEGFB, CD40LG and PDCD1 (PD1; Fig. 2b–d,f,g). Immunosuppressive macrophage M2 enrichment was also significantly associated with poor outcomes in the mFFX post-CTX group (Fig. 2e). Further, and consistent with earlier studies, ‘deserted’ TME signatures were significantly upregulated in post-CTX mFFX samples, suggesting that neoadjuvant mFFX preferentially enriched for matrix-rich chemoprotective TMEs (Fig. 2c)^20^.

### Transcriptomic subtypes and outcomes post-CTX

Initial indications have suggested that transcriptomic subtypes can prognosticate in individuals with resectable and metastatic disease^24,25^. To determine whether PDAC subtypes prognosticate in PDAC-HD samples, we performed subtyping analysis using Collisson, Moffitt, Bailey or Notta subtyping schemes (Fig. 1e, Extended Data Fig. 1b,c and Supplementary Table 4)^24,26–28^.

Subtyping analysis of chemo-naive PDAC-HD samples demonstrated that Moffitt and Notta subtypes were prognostic for PDAC-HD chemo-naive samples (Fig. 1e and Extended Data Fig. 1b,c). Consistent with earlier findings, Bailey, Collison and Notta subtypes exhibited considerable overlap with the Moffitt classical and basal-like subtypes (Extended Data Fig. 1b). Notably, 71% (*n* = 45) of the PDAC-HD chemo-naive samples were identified as belonging to the classical subtype, whereas the remaining 29% (*n* = 19) were identified as basal-like.

Subtyping analysis of post-CTX resected PDAC-HD samples demonstrated that both classical and basal-like subtypes persisted after therapy (Fig. 1c). In comparison to chemo-naive PDAC-HD samples, the basal-like subtype was found to be significantly overrepresented in post-CTX samples compared to the classical subtype (*P* < 0.001, chi-squared test, two sided; Fig. 1d). Enrichment of the basal-like subtype in post-CTX samples was independent of clinical stage at time of treatment (Extended Data Fig. 1d). Survival analysis revealed that although Moffitt subtypes were prognostic for chemo-naive PDAC-HD samples (log-rank *P* = 0.016), they did not discriminate between good and poor outcomes in PDAC-HD post-CTX samples (log-rank *P* = 0.38; Fig. 1e and Extended Data Fig. 1c). Similarly, the Bailey (log-rank *P* = 0.43), Collisson (log-rank *P* = 0.68) and Notta (log-rank *P* = 0.061) subtyping schemes did not predict outcome in post-CTX PDAC-HD groups. To corroborate these findings, purity independent subtyping of tumors (PurIST) was applied to the PDAC-HD cohort^25^. Although high PurIST scores exhibited significant overlap with the Moffitt basal subtype, high and low PurIST scores similarly did not prognosticate in PDAC-HD post-CTX samples (log-rank *P* = 0.65: high 13 versus low 15). These findings, although representative of the participants included in this study, will require further validation in suitably matched independent cohorts.

### GATA6^+^ and KRT17^+^ cell phenotypes persist post-CTX

**Fig. 3:**
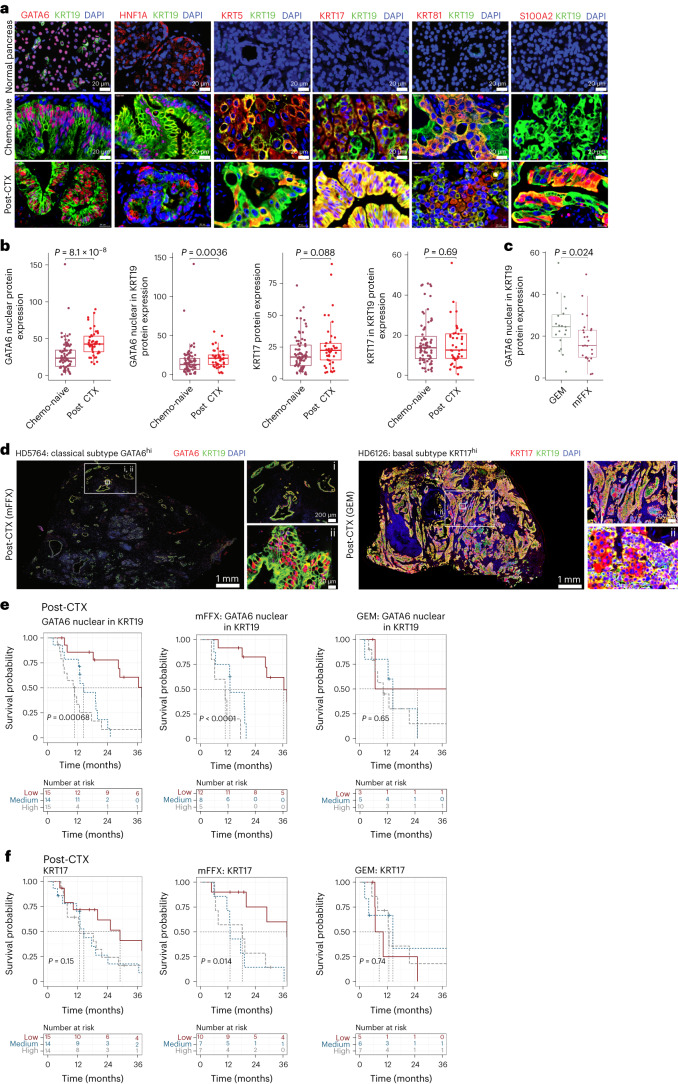
Classical and basal biomarker analysis of chemo-naive and post-CTX samples using multiplexed IF. **a**, Multiplexed IF images of representative normal (*n* = 9), chemo-naive (*n* = 77) and post-CTX (*n* = 45) samples stained with GATA6 (red), HNF1A (red), KRT5 (red), KRT17 (red), KRT81 (red), S100A2 (red) and KRT19 (green) antibodies. **b**, Box plots showing relative whole-section protein expression of the indicated biomarkers in chemo-naive (*n* = 77) and post-CTX (*n* = 45) samples. Biomarker protein expression is considered alone or in the context of KRT19 coexpression. Kruskal–Wallis rank-sum test (two-sided) *P* values are provided at the top of each plot. **c**, Box plot showing relative whole-section protein expression of nuclear GATA6 in KRT19^+^ cells between GEM (*n* = 19) and mFFX (*n* = 25) post-CTX samples. The Kruskal–Wallis rank-sum test (two-sided) *P* value is provided at the top. **d**, Multiplexed IF images of representative classical and basal post-CTX samples stained with GATA6 (red), KRT17 (red) and KRT19 (green) antibodies. Representative images are presented in rows, with the leftmost image showing the entirety of the imaged region. The top right image and bottom right image show selected regions (i and ii) at increased magnification. **e**,**f**, Kaplan–Meier survival analysis for high, medium and low GATA6 and KRT17 protein expression tertiles in post-CTX PDAC-HD samples. Kaplan–Meier survival analyses for post-CTX samples representing combined (GEM and mFFX) treatment, GEM alone or mFFX alone are shown. Participant numbers for each group are provided under ‘Numbers at risk’. A log-rank *P* value of ≤0.05 is considered significant. All box plots show the median (line), the interquartile range (IQR) between the 25th and 75th percentiles (box) and 1.5× the IQR ± the upper and lower quartiles. *P* values were not adjusted for multiple testing. Source data

The persistence of subtype-specific programs in post-CTX samples suggested that neoplastic cell phenotypes may contribute to outcomes following neoadjuvant chemotherapy. To characterize neoplastic cell phenotypes in PDAC-HD samples, we performed immunofluorescence (IF) staining using validated antibodies to GATA6, HNF1A, KRT5, KRT17, KRT81 and S100A2 (Fig. 3a–c and Supplementary Table 13). Biomarker expression was determined alone or in the context of KRT19 coexpression, a ductal biomarker that is significantly upregulated in atypical ductal cells and PDAC^16^ (Fig. 3a–c).

GATA6 is broadly considered a surrogate marker of the classical-like subtype^27,29–34^. GATA6 protein expression by immunohistochemistry is predictive of better survival with 5-fluorouracil/folinic acid in the adjuvant (chemo-naive) setting but not with adjuvant GEM^33^. KRT5 (ref. ^35^), KRT17 (ref. ^36^), KRT81 (ref. ^37^) and S100A2 (ref. ^17^) are biomarkers associated with basal/squamous cell phenotypes (although these biomarkers may not represent the full spectrum of basal/squamous cell states).

**Extended Data Fig. 3:**
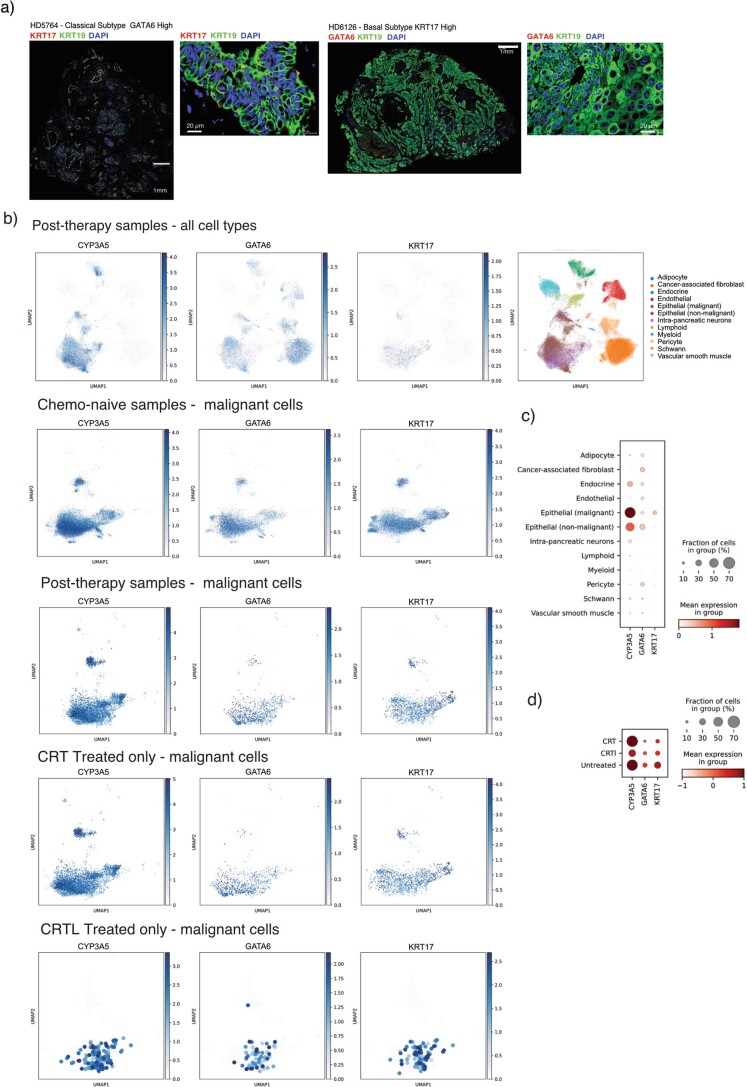
Multiplexed IF and single nuclei RNAseq identifies complex intratumor heterogeneity. **a**) Multiplexed IF showing the spatial expression of KRT17 and GATA6 in Classical subtype GATA6 High and Basal subtype KRT17 High samples, respectively. These images correspond to whole tissue sections shown in Fig. 2d. Scale bars are shown in the images. **b**) Reanalysis of single nuclei RNAseq data first published in Hwang et al.^16^ demonstrating the presence of GATA6, KRT17 and CYP3A5 co-expressing cells in chemo-naïve samples that persist following chemotherapy and nearly always radiotherapy. UMAP embeddings of single nuclei gene expression profiles representing the indicated cell types in chemo-naïve and post-therapy patient samples. CRT denotes chemoradiotherapy (FOLFIRINOX as the major chemotherapy regimen) and CRTL denotes chemoradiotherapy (FOLFIRINOX as the major chemotherapy regimen) plus losartan. **c**) Dot plot showing the relative enrichment of GATA6, CYP3A5, and KRT17 in cell types comprising post-CRT samples. **d**) Dot plot showing the relative enrichment of GATA6, CYP3A5, and KRT17 in chemo-naïve (untreated) and post-CRT or post-CRTL patient samples. Source data

GATA6 protein expression was determined by IF in chemo-naive and post-CTX PDAC-HD samples (Fig. 3b). This analysis demonstrated that the percentage of nuclear GATA6 expression was significantly elevated post-CTX (considering GEM and mFFX samples together) compared to the chemo-naive group. This trend of increased GATA6 expression was also observed when the percent expression of nuclear GATA6 in KRT19^+^ cells was considered. Stratifying post-CTX samples by chemotherapy regimen demonstrated that GATA6/KRT19 expression was significantly higher in GEM samples than in the mFFX group, suggesting that chemotherapy regimen may influence the number of GATA6-expressing cells (Fig. 3c). Imaging of representative classical GATA6^hi^KRT17^low^ and basal-like KRT17^hi^GATA6^low^ samples clearly demonstrated dominant biomarker expression in post-CTX samples (Fig. 3d and Extended Data Fig. 3a). Taken together, these data revealed that GATA6^hi^ and KRT17^hi^ cell phenotypes persisted after chemotherapy.

**Extended Data Fig. 4:**
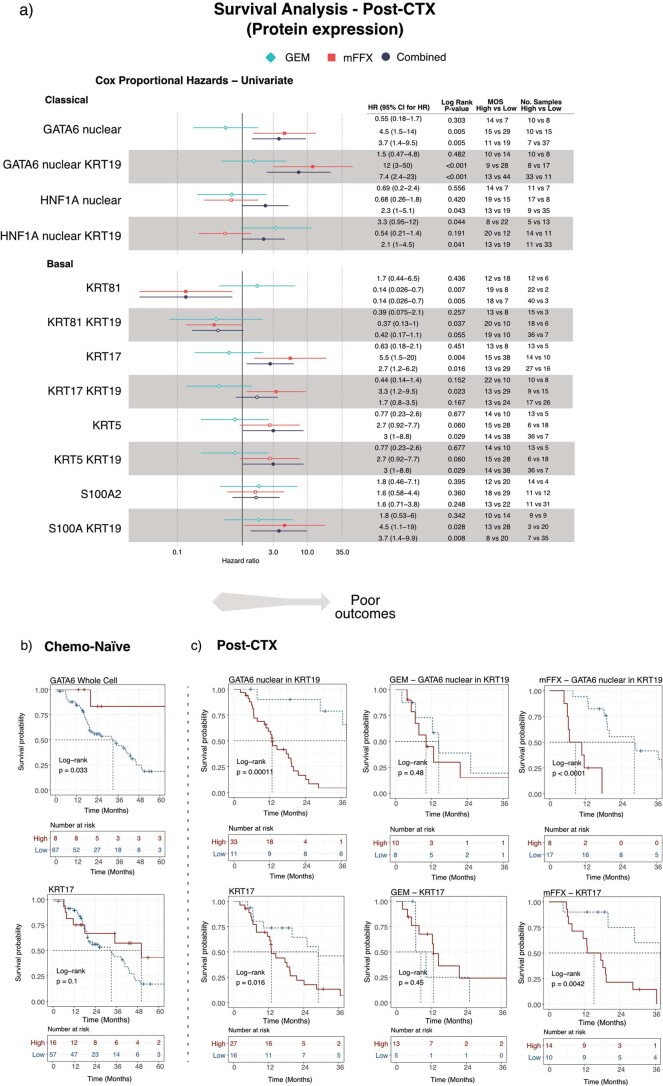
GATA6^Hi^ and KRT17^Hi^ persister phenotypes are associated with poor patient outcomes in mFOLFIRINOX but not gemcitabine post-CTX patient samples. **a**) Forest plot and associated table of discovery analyses showing univariate cox proportional hazards for neoadjuvant post-CTX with patient samples dichotomized with high and low protein biomarker expression. Analyses are shown for combined GEM and mFFX (black) (n = 44), GEM alone (cyan) (n = 18) and mFFX alone (red) (n = 25) post-CTX patient samples. Hazard Ratios (HR) are shown on the plot as the central measure (symbol) with 95% Confidence Intervals (CI) shown as bars. Log-rank *P*-values (two-sided) and Median Overall Survival (MOS) for high and low expressing groups are provided for each comparison. Log-rank *P*-values were not adjusted for multiple testing. **b**) Kaplan Meier survival analysis for dichotomized High and Low GATA6 and KRT17 protein expression in chemo-naïve PDAC-HD samples. **c)** Kaplan Meier survival analysis for dichotomized High and Low GATA6 and KRT17 protein expression in post-CTX PDAC-HD samples. Kaplan Meier survival analysis of post-CTX patient samples representing combined (GEM and mFFX), GEM alone or mFFX alone is shown. For panels b) and c), patient numbers for each group are provided under ‘Numbers at risk’. Log Rank P-value ≤ 0.05 is considered significant. Log-rank *P*-values were not adjusted for multiple testing. Source data

To determine whether persistent GATA6^hi^ classical and KRT17^hi^ basal-like phenotypes in resected post-CTX samples are associated with outcomes, we categorized IF levels according to robust tertiles of protein expression and performed a survival analysis (Fig. 3e,f). High GATA6 protein expression in post-CTX samples was associated with significantly worse outcomes (Fig. 3e). To further understand the relationship between GATA6 protein expression and neoadjuvant chemotherapy, we examined whether the association between GATA6^hi^ expression and poor outcome was common or unique to either GEM or mFFX neoadjuvant chemotherapy. Importantly, we found that GATA6^hi^ expression was associated with significantly worse outcomes after mFFX, but not GEM, neoadjuvant chemotherapy (Fig. 3e). A similar assessment of KRT17 IF expression tertiles demonstrated that KRT17^hi^ samples were significantly associated with worse outcomes after mFFX, but not GEM, neoadjuvant chemotherapy (Fig. 3f). Exploratory survival analysis using basal/squamous biomarkers KRT5, KRT81 and S100A2 revealed additional significant associations, although validation in independent cohorts will be required (Extended Data Fig. 4; please note that exploratory univariate analyses were not corrected for multiple testing). Collectively, these data demonstrate that GATA6^hi^ and KRT17^hi^ phenotypes persist post-CTX and contribute to poor outcomes in individuals after neoadjuvant chemotherapy.

### Complex neoplastic heterogeneity defines post-CTX samples

**Extended Data Fig. 5:**
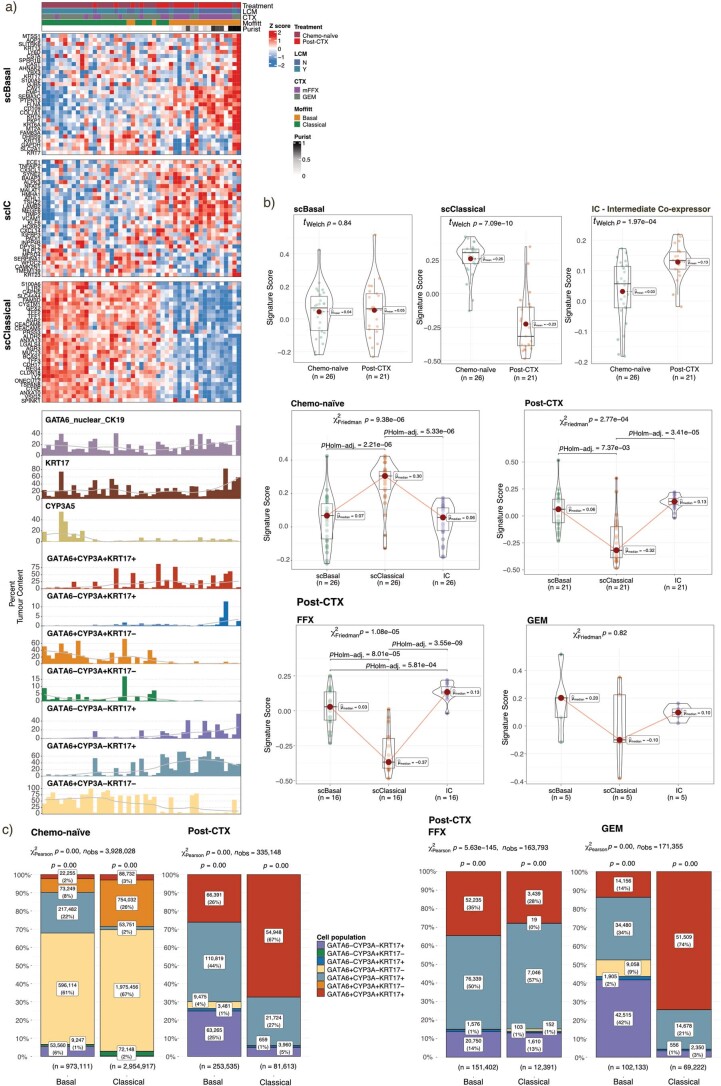
Intermediate co-expressor (IC) ‘Hybrid’ states are significantly enriched in post-CTX samples. **a**) Top panel, heatmap showing the expression of gene signatures derived from single cell analyses that define distinct Classical (scClassical), Basal (scBasal) and Intermediate co-expressor (IC) cell phenotypes. Patient samples are ordered according to increasing scBasal ssGSEA enrichment scores. Bottom panel, bar charts showing the percent tumor enrichment of GATA6/CYP3A/KRT17 cell populations as determined by multiplexed IF. Patient samples in top panel and bottom panel are identical and similarly ordered. A LOESS regression line has been added to each bar plot. **b**) Box plots showing the enrichment of scClassical, scBasal and scIC signature scores in chemo-naïve and post-CTX patient samples. Sample numbers (n) shown at the bottom of each plot. Two-sided Welch’s t-test was performed to determine significance between treatment groups. Welch’s *P*-values were not corrected for multiple testing. Two-sided Friedman rank sum test was performed to determined significance between signature score in indicated treatment group. Friedman *P*-values were adjusted for multiple testing. Boxplots show the median (line), the interquartile range (IQR) between the 25th and 75th percentiles (box) and 1.5× the IQR ± the upper and lower quartiles. **c**) Bar stat plots showing the number of GATA6/KRT17/CYP3A ‘hybrid’ cells observed (n) in Moffitt subtypes Basal (n = 20) and Classical (n = 27). A Pearson χ^2^-test of independence (two-sided) is provided at the top of each plot and *P*-values from a one sample proportions test (two-sided) are displayed on the top of each bar. *P*-values were not adjusted for multiple testing. Source data

Classical and basal-like subtype cell populations may coexist intratumorally^17,28^. Single-cell RNA-seq of chemo-naive and biopsied liver metastases has identified hybrid or IC neoplastic cell states that share biomarkers common to both the classical-like and basal-like/squamous subtypes, namely GATA6 and KRT17 (ref. ^17^). Enrichment analysis using gene signatures for single-cell classical (scClassical), scBasal and scIC cell states demonstrated that the scIC cell state is preferentially enriched in post-CTX samples (Extended Data Fig. 5a,b), suggesting that neoadjuvant chemotherapy may promote ‘hybrid’ cell states.

**Fig. 4:**
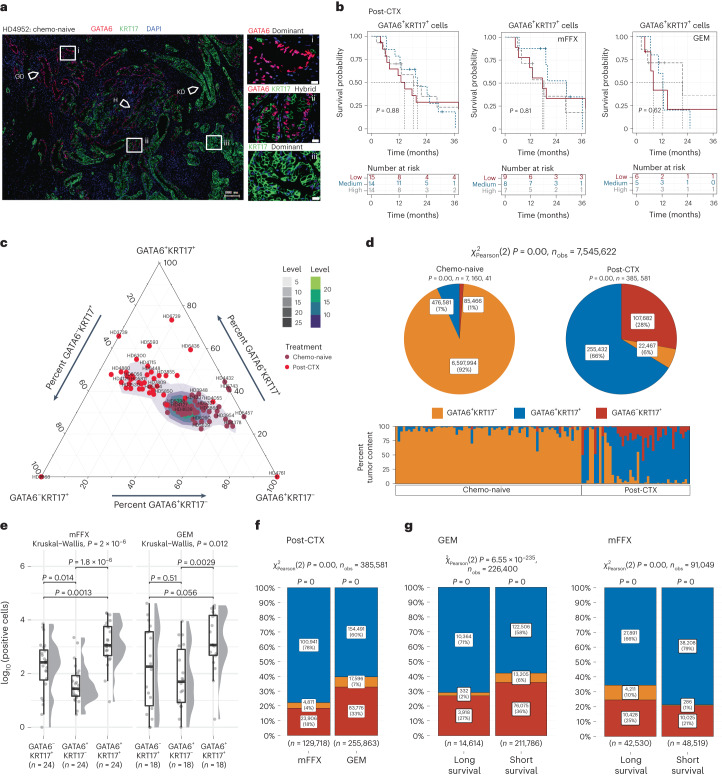
Multiplexed GATA6 and KRT17 IF identifies complex intratumor heterogeneity in post-CTX samples. **a**, Multiplexed IF images of post-CTX samples stained with GATA6 (red), KRT17 (green) and DAPI (blue). Left, whole-section images; scale bar, 200 μm. Arrows demarcate foci representing dominant GATA6 staining (GD), dominant KRT17 staining (KD) and ‘hybrid’ GATA6^+^KRT17^+^ (H) staining. Regions of interest (ROIs) demarcated by white boxes and labeled by i, ii or iii are shown at higher magnification on the right; scale bar, 20 μm. **b**, Kaplan–Meier survival analysis for high, medium and low ‘hybrid’ GATA6^+^KRT17^+^ protein expression tertiles in post-CTX PDAC-HD. Participant numbers for each group are provided under ‘Numbers at risk’. A log-rank *P* value of ≤0.05 is considered significant. **c**, Ternary plot showing the percent tumor content of GATA6 and KRT17 cell populations in chemo-naive (*n* = 69) and post-CTX (*n* = 42) patients. **d**, Pie stat plots and bar chart showing enrichment of GATA6^hi^KRT17^hi^ ‘hybrid’ persister phenotypes in chemo-naive (*n* = 69) and post-CTX (*n* = 42) samples. Pearson chi-squared test of independence (two sided) is highly significant (*P* = 0) given a large sample size (*n*_obs_ = 7,545,622 cells). **e**, Box plots showing the relative protein expression of GATA6/KRT17 cell phenotypes post-CTX GEM (*n* = 18) or after mFFX (*n* = 24). Kruskal–Wallis rank-sum test (two-sided) *P* values are provided in the plot. Box plots show the median (line), the IQR between the 25th and 75th percentiles (box) and 1.5× the IQR ± the upper and lower quartiles. **f**, Bar stat plot showing the percentage of GATA6/KRT17 cell phenotypes in post-CTX samples (*n* = 42). **g**, Bar stat plots showing the percentage of GATA6/KRT17 cell phenotypes enriched in samples treated preoperatively with either GEM (*n* = 18) or mFFX (*n* = 24) and associated with long and short survival. With respect to bar stat plots, Pearson chi-squared test of independence (two sided) is highly significant (*P* = 0) given the large sample sizes (*n*_obs_ = 385,581 cells for GEM/mFFX in **f**, and *n*_obs_ = 226,400 cells for GEM and *n*_obs_ = 91,049 cells for mFFX in **g**). The *P* values from a one-sample proportions test (two sided) are displayed on the top of each bar. *P* values were not adjusted for multiple testing. Source data

To assess whether GATA6^hi^ and KRT17^hi^ persister cell phenotypes are mutually exclusive or coexist in PDAC-HD samples, we performed multiplexed co-staining (colocalization) for GATA6, KRT17 and KRT19 (Fig. 4a and Supplementary Table 9). This analysis revealed complex patterns of intratumoral expression. These included individual samples exhibiting dominant GATA6^hi^KRT17^low^ or dominant KRT17^hi^GATA6^low^ expression and samples with interspersed mosaic GATA6^hi^KRT17^low^ and KRT17^hi^GATA6^low^ expression foci within the same tissue section. A subset of individual samples also exhibited hybrid staining with GATA6 and KRT17 expressed in the same cells. Most strikingly, a number of samples exhibited a gradient of biomarker expression wherein dominant GATA6^hi^KRT17^low^, GATA6^hi^KRT17^hi^ hybrid and dominant KRT17^hi^ GATA6^low^ expression foci were arranged serially within the same tissue section (Fig. 4a).

Deconvolution of biomarker intensities in whole-tissue sections allowed us to count individual cells according to biomarker expression with *n*_obs_ = 7,545,622 cells designated as GATA6^+^KRT17^–^, GATA6^–^KRT17^+^ or GATA6^+^KRT17^+^ (Fig. 4c,d). Comparison of cell-type abundance between chemo-naive and post-CTX samples revealed that GATA6^+^KRT17^–^ cells are enriched in chemo-naive samples, while GATA6^+^KRT17^+^ and GATA6^–^KRT17^+^ cell populations are preferentially enriched post-CTX (Fig. 4c,d). Samples treated preoperatively with mFFX exhibited significantly greater numbers of GATA6^+^KRT17^+^ cells than those treated preoperatively with GEM (Fig. 4e,f).

Survival analysis demonstrated no significant association between GATA6^+^KRT17^+^ cell enrichment and outcome in either the chemo-naive or post-CTX groups (Fig. 4b). To disentangle the relationship between participant outcomes and GATA6/KRT17 cell phenotypes, post-CTX samples were dichotomized using GATA6 expression tertiles, as described earlier, and GATA6/KRT17 cell populations were compared. In the context of mFFX neoadjuvant chemotherapy, samples with low GATA6 expression (associated with longer survival) had increased numbers of GATA6^+^KRT17^–^ cells, whereas samples with high GATA6 expression (associated with shorter survival) had increased numbers of GATA6^+^KRT17^+^ hybrid cells but significantly fewer GATA6^+^KRT17^–^ cells (Fig. 4g). GATA6 protein expression was not prognostic for GEM neoadjuvant chemotherapy, and we failed to observe a similar shift toward higher GATA6^+^KRT17^+^ hybrid cell numbers or near loss of GATA6^+^KRT17^–^ cells in the GATA6^hi^ samples treated preoperatively with GEM. These findings suggest that poor outcomes associated with GATA6^hi^ and KRT17^hi^ expression in post-CTX samples involve a higher relative enrichment of GATA6^+^KRT17^+^ and GATA6^–^KRT17^+^ cells and near loss of GATA6^+^KRT17^–^ cells.

### Drug-tolerant persister cell phenotypes

**Fig. 5:**
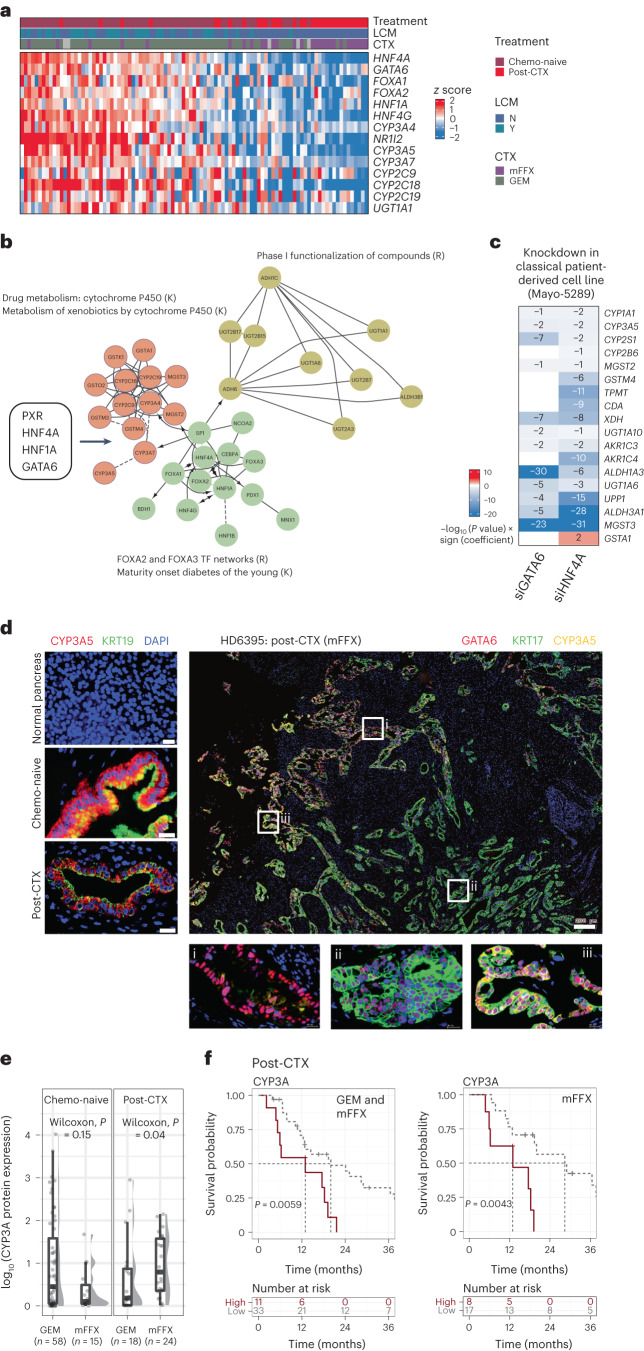
CYP3A protein expression in mFFX post-CTX samples is associated with patient outcome. **a**, Heat map showing mRNA expression of coexpressed genes associated with drug metabolism and phase I functionalization of compounds. **b**, Network of coexpressed genes that are enriched in classical-like samples. Gene nodes (circles) are colored according to their annotated molecular functions. K denotes annotated KEGG pathways, and R denotes annotated REACTOME pathways. Transcription factors that regulate the network of genes are shown in the adjacent box. **c**, RNA-seq reanalysis of *GATA6* and *HNF4A* siRNA knockdown experiments performed in a classical human-derived cell line (*n* = 3 control; *n* = 3 siRNA) as described in Brunton et al.^31^. Heat map values represent –log_10_ (*P* values) × sign (coefficient). Blue color indicates downregulation in siRNA-treated cells. *P* values represent the significance of a two-sided Wald test and were adjusted for multiple testing. **d**, Left, multiplexed IF images of representative normal, chemo-naive and post-CTX PDAC-HD samples showing spatial expression of CYP3A relative to KRT19-expressing cells. Right, multiplexed IF images of a representative post-CTX sample analyzed with antibodies to GATA6 (red), KRT17 (green) and CYP3A (yellow); scale bar, 200 μm (whole-section image). ROIs demarcated by white boxes and labeled by i, ii or iii are shown at higher magnifications; scale bar, 20 μm. ROIs represent dominant GATA6 staining (i), dominant KRT17 staining (ii) and ‘hybrid’ GATA6^+^KRT17^+^CYP3A^+^ (iii) staining. **e**, Box plots showing protein expression by IF of CYP3A according to treatment. Kruskal–Wallis rank-sum test (two-sided) *P* values are shown on the plots. Box plots show the median (line), the IQR between the 25th and 75th percentiles (box) and 1.5× the IQR ± the upper and lower quartiles. *P* values were not adjusted for multiple testing. **f**, Kaplan–Meier survival analysis for high (highest 25% of IF values) and low CYP3A protein expression (remainder of IF values) in post-CTX PDAC-HD samples combined (GEM and mFFX) or in mFFX samples alone. Participant numbers for each group are provided under ‘Numbers at risk’. A log-rank *P* value of ≤0.05 is considered significant. *P* values were not adjusted for multiple testing. Source data

Network analyses of GPs enriched in chemo-naive samples identified a subnetwork of coexpressed genes that comprised key pancreatic transcription factors GATA6, HNF4A, HNF1A, FOXA2 and FOXA3 and genes involved in drug metabolism, that is, CYP450 enzymes and phase I functionalization of compounds (Fig. 5a,b). The coexpression of this subnetwork of genes, while highly expressed in chemo-naive samples, was retained in a subset of post-CTX samples (Fig. 5a). Of particular interest was the identification of CYP450 family genes (*CYP3A4*, *CYP3A5* and *CYP2C9*), which were coexpressed with transcription factors GATA6, HNF4A and NR1I2 (also known as pregnane X receptor (PXR); Fig. 5a,b). Transcription factors HNF4A and NR1I2 (PXR) have been shown to regulate both steady-state and substrate-induced expression of CYP3A5 in both classical and basal-like pancreatic cancer cell lines^37^. A reanalysis of existing data^31^ demonstrated that GATA6 and HNF4A are required for the expression of not only CYP3A5 but also several other genes involved in drug detoxification (Fig. 5c).

The CYP3A subfamily (encoded by 4 genes, *CYP3A4*, *CYP3A5*, *CYP3A7* and *CYP3A43*) has been shown to metabolize common cytotoxic chemotherapeutics, including irinotecan, paclitaxel, docetaxel, anthracyclines, vinca alkaloids and tyrosine kinase inhibitors, such as erlotinib and gefitinib, in the liver and intestinal epithelia^37^. The contribution of these genes to extrahepatic drug tolerance is poorly understood^38–41^. Recent evidence, however, suggests that tumor cell-intrinsic expression of CYP3A5 may underpin intrinsic drug resistance in colorectal cancer and PDAC^37,42^. CYP3A5 activity has been associated with resistance to paclitaxel in pancreatic cancer cell lines^37^ and resistance to irinotecan in colorectal cancer^42^. Given that irinotecan, a constituent of mFFX, is a substrate of CYP3A family proteins, we surmised that CYP3A family proteins might underpin the persistence of drug-resistant GATA6^hi^ phenotypes in mFFX post-CTX samples.

Given the tight relationship between *CYP3A4*/*CYP3A5* and *CYP3A7* gene expression, we stained for CYP3A5 protein (referred to herein as CYP3A) as a biomarker reflecting expression of the broader CYP3A network (Fig. 5d). CYP3A protein expression was initially assessed in normal pancreatic donor tissue, chemo-naive and post-CTX PDAC-HD samples (Fig. 5d, left, and Supplementary Table 5). Importantly, we observed a significant increase in CYP3A protein expression in samples treated preoperatively with mFFX, but not GEM, further supporting the role of CYP3A as an important mediator of mFFX drug resistance (Fig. 5e).

**Extended Data Fig. 6:**
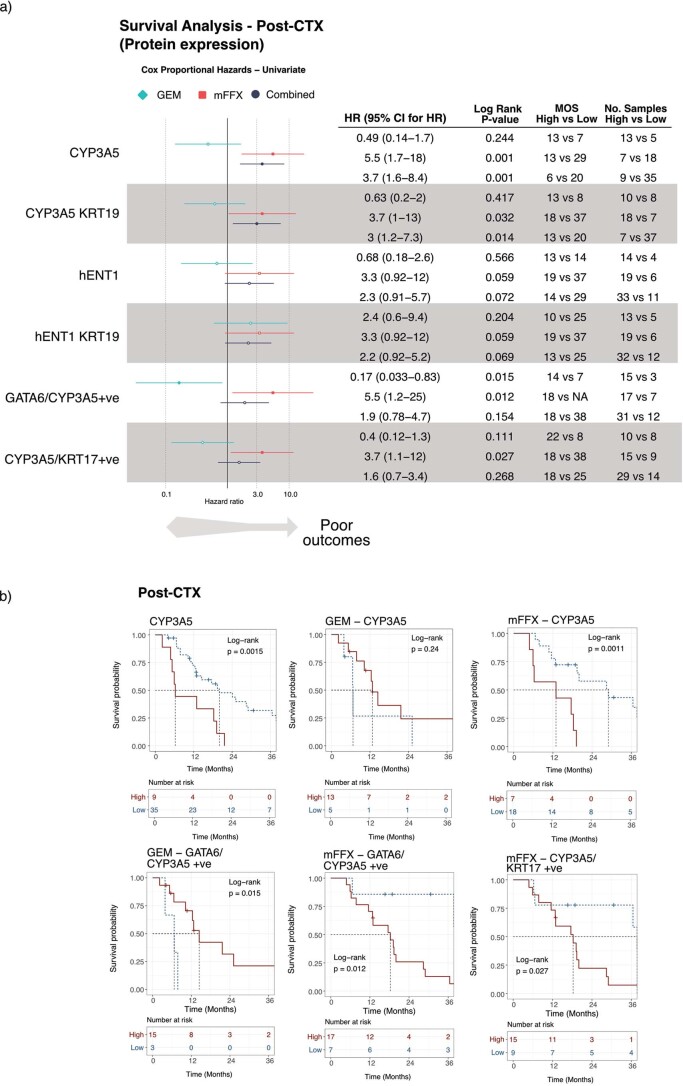
CYP3A^Hi^ persister phenotypes are associated with poor patient outcomes in mFFX but not GEM post-CTX patient samples. **a**) Forest plot and associated table of discovery analyses showing univariate cox proportional hazards for post-CTX patient samples with the indicated dichotomized High and Low protein biomarker expression. Univariate analyses have not been corrected for multiple testing. Analyses are shown for combined GEM and mFFX (black) (n = 44), GEM alone (cyan) (n = 18) and mFFX alone (red) (n = 25) post-CTX patient samples. Hazard Ratios (HR) are shown on the plot as the central measure (symbol) with 95% Confidence Intervals (CI) shown as bars. Log-rank *P*-values (two-sided) and Median Overall Survival (MOS) for high and low expressing groups are provided for each comparison. Log-rank *P*-values were not adjusted for multiple testing. **b**) Kaplan Meier survival analysis for dichotomized High and Low CYP3A, ‘hybrid’ CYP3A/KRT17^+ve^ and CYP3A/KRT17^+ve^ persister phenotypes in post-CTX PDAC-HD samples. Kaplan Meier survival analysis of post-CTX patient samples representing combined (GEM and mFFX), GEM alone or mFFX alone is shown. Patient numbers for each group are provided under ‘Numbers at risk’. Log Rank P-value ≤ 0.05 is considered significant. Log-rank *P*-values were not adjusted for multiple testing. Source data

To determine whether CYP3A protein expression was prognostic in PDAC-HD samples, we performed a survival analysis using dichotomized CYP3A values representing high (highest 25% of IF values; *n* = 11 participants) and low (remainder of IF values; *n* = 33 participants) expression. This expression cutoff was used, as CYP3A values were negatively skewed (Fig. 5f and Extended Data Fig. 6). In the post-CTX group, high CYP3A protein expression was associated with significantly worse outcomes. Mirroring the results obtained for GATA6, high CYP3A protein expression in mFFX, but not GEM, was associated with significantly worse outcomes. Exploratory analysis using antibodies specific to hENT1, an established biomarker of GEM resistance in PDAC, found no significant association between hENT1 expression and participant outcome in either setting, although validation in independent cohorts will be required^43–45^ (Extended Data Fig. 6; as before, exploratory univariate analyses were not corrected for multiple testing).

### CYP3A expression is associated with worse outcome

**Fig. 6:**
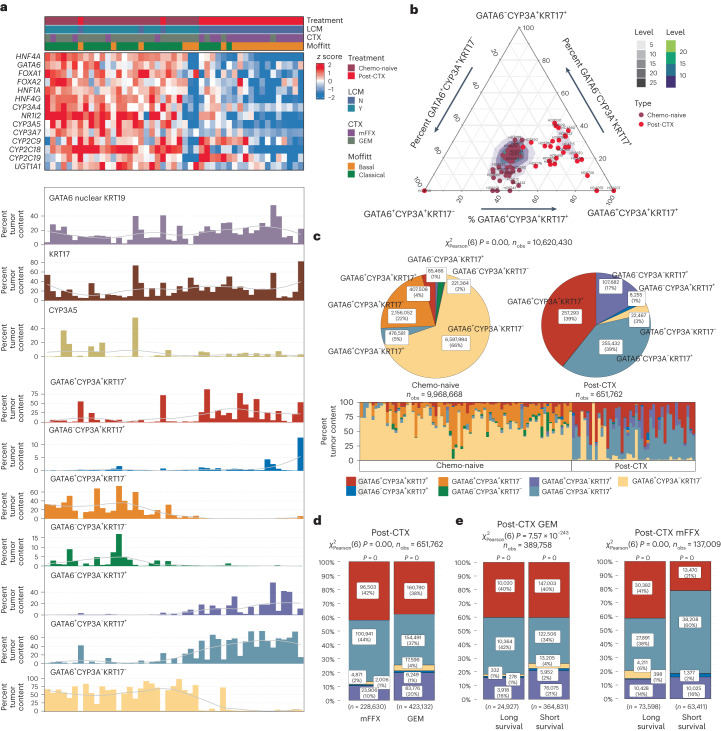
Multiplexed GATA6, KRT17 and CYP3A IF identifies CYP3A ‘hybrid’ persister phenotypes enriched in post-CTX samples. **a**, Top, heat map showing the relative mRNA expression of coexpressed genes associated with drug metabolism and phase I functionalization of compounds. Bottom, bar charts showing the percent tumor enrichment of GATA6/CYP3A/KRT17 cell populations as determined by multiplexed IF. Samples used to generate the data in the top and bottom are identical (*n* = 47) and are similarly ordered. A LOESS regression line has been added to each bar plot. **b**, Ternary plot showing the percent tumor content of GATA6, CYP3A and KRT17 cell populations in chemo-naive (*n* = 69) and post-CTX (*n* = 42) samples. Post-CTX samples show an enrichment for CYP3A^+^ ‘hybrid’ persister phenotypes. **c**, Pie stat plots and bar chart showing significant enrichment of GATA6/CYP3A/KRT17 ‘hybrid’ persister phenotypes in chemo-naive (*n* = 69) and post-CTX (*n* = 42) samples. Pearson chi-squared test of independence (two sided) is highly significant (*P* = 0) given a large sample size (*n*_obs_ = 10,620,430 cells). **d**, Bar stat plot showing the percentage of GATA6/CYP3A/KRT17 ‘hybrid’ cell phenotypes in post-CTX samples treated with either GEM (*n* = 18) or mFFX (*n* = 24). **e**, Bar stat plots showing the percentage of GATA6/CYP3A/KRT17 ‘hybrid’ cell phenotypes enriched in samples treated preoperatively with either GEM (*n* = 18) or mFFX (*n* = 24) and associated with long and short survival. For bar stat plots, Pearson chi-squared test of independence (two sided) is highly significant (*P* = 0) given the large sample sizes (*n*_obs_ = 651,762 cells for GEM/mFFX in **d**, and *n*_obs_ = 389,758 cells for GEM and *n*_*o*bs_ = 137,009 cells for mFFX in **e**). The *P* values from a one-sample proportions test (two sided) are displayed on the top of each bar. *P* values were not adjusted for multiple testing. Source data

**Extended Data Fig. 7:**
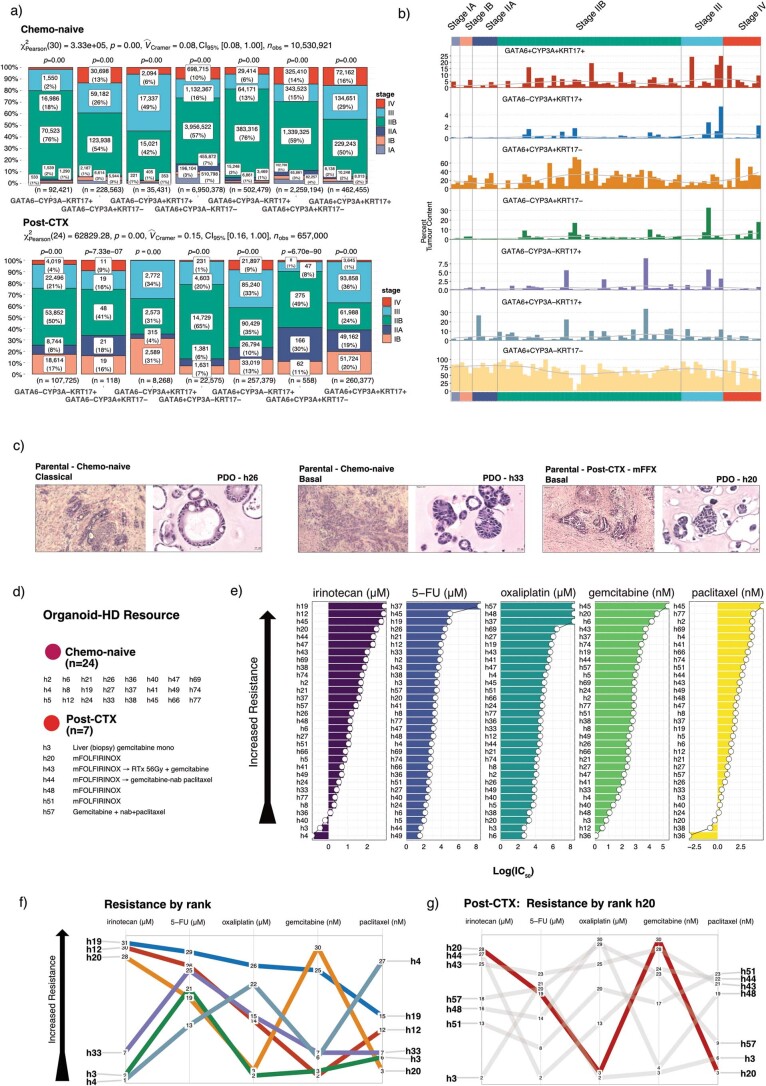
AJCC (8^th^ Edition) Stage is associated with GATA6/ CYP3A/KRT17 ‘hybrid’ cell enrichment in chemo-naïve PDAC-HD samples. **a**) Bar stat plots showing the number of GATA6/KRT17/CYP3A ‘hybrid’ cells observed in different AJCC stages for both chemo-naïve (n = 69) and post-CTX samples (n = 42). As above, a Pearson χ^2^-test of independence (two-sided) is provided at the top of each plot and *P*-values from a one sample proportions test (two-sided) are displayed on the top of each bar. Number of observations (n) cells are provided at the bottom of each bar. P-values were not adjusted for multiple testing. **b**) Bar plot showing the percent tumor content of GATA6/KRT17/CYP3A ‘hybrid’ cells in chemo-naïve samples (n = 69) for each AJCC stage. A LOESS regression line has been added to each bar plot. **c)** Hematoxylin and Eosin (H&E) stains of representative patient samples showing the histology of the parental tissue and matched PDO. **d**) Table describing PDAC-HD organoids used in this study. **e)** Pharmacotyping of PDOs showing heterogeneity of chemotherapy response. PDOs are ranked by increasing Log (IC50) values for the indicated drug treatments and concentrations. **f)** Line plot showing the heterogeneity of chemotherapy responses for PDOs resistant and susceptible to irinotecan. **g**) Line plot showing chemotherapy responses for PDAC-HD PDO h20 which was generated from a patient sample that had received mFFX pre-operatively. Numbers in line plots represent the Log (IC50) rank for the indicated treatment. Source data

To determine whether persistent GATA6/KRT17 cell types coexpressed CYP3A, we performed multiplexed CYP3A, GATA6 and KRT17 IF on PDAC-HD samples. As before, this analysis identified complex spatial patterns of CYP3A, GATA6 and KRT17 coexpression (Fig. 5d, right). Deconvolution of whole-tissue sections from chemo-naive samples (*n*_obs_ = 9,968,668 cells) identified distinct subpopulations of CYP3A-expressing cells (GATA6^+^CYP3A^+^KRT17^+^, GATA6^+^CYP3A^+^KRT17^–^, GATA6^–^CYP3A^+^KRT17^+^ and GATA6^–^CYP3A^+^KRT17^–^) and CYP3A-non-expressing cells (GATA6^+^CYP3A^–^KRT17^–^ and GATA6^–^CYP3A^–^KRT17^+^; Fig. 6a–c). GATA6^+^CYP3A^–^KRT17^–^ cells (66%) were found to make up the highest relative percentage in chemo-naive samples, followed by GATA6^+^CYP3A^+^KRT17^–^ (22%), GATA6^+^CYP3A^–^KRT17^+^ (5%), GATA6^+^CYP3A^+^KRT17^+^ (4%), GATA6^–^CYP3A^+^KRT17^–^ (2%) and GATA6^–^CYP3A^–^KRT17^+^ (1%) cells. Importantly, the enrichment of distinct cell populations in chemo-naive samples was associated with AJCC tumor stage^19^ (Extended Data Fig. 7a,b). GATA6^+^CYP3A^–^KRT17^–^ cells exhibited the highest percent expression in early-stage tumors (stages IA, IB, IIA and IIB), whereas CYP3A-expressing cells, including GATA6^–^CYP3A^+^KRT17^–^, GATA6^–^CYP3A^+^KRT17^+^ and GATA6^+^CYP3A^+^KRT17^+^, had the highest relative enrichment in later-stage tumors (stages IIB, III and IV).

Analysis of post-CTX samples (*n*_obs_ = 651,762 cells) revealed that GATA6^+^CYP3A^+^KRT17^+^ (39%), GATA6^+^CYP3A^–^KRT17^+^ (39%) and GATA6^–^CYP3A^–^KRT17^+^ (17%) cells had the highest percent enrichment (Fig. 6c). By contrast, the percentage of GATA6^+^CYP3A^–^KRT17^–^ cells was significantly reduced in the post-CTX group, which was consistent with our early findings. Integration of RNA-seq and multiplexed IF data further demonstrated that the differential expression of drug-metabolizing genes between chemo-naive and post-CTX samples was associated with the enrichment of different GATA6/CYP3A/KRT17-expressing subpopulations (Fig. 6a and Extended Data Fig. 5). Chemo-naive samples exhibiting high *CYP3A5* mRNA expression and predominant scClassical transcriptomic profiles were associated with a higher percent enrichment of GATA6^+^CYP3A^+^KRT17^–^, GATA6^–^CYP3A^+^KRT17^–^ and GATA6^+^CYP3A^–^KRT17^–^ cells. By contrast, downregulation of *CYP3A5* mRNA expression and predominant scIC transcriptomic profiles were associated with the emergence of GATA6^+^CYP3A^+^KRT17^+^, GATA6^–^CYP3A^+^KRT17^+^, GATA6^–^CYP3A^–^KRT17^+^ and GATA6^+^CYP3A^–^KRT17^+^ cells in post-CTX samples (Extended Data Fig. 5a). These results, which are supported by recent single-cell RNA-seq studies (Extended Data Fig. 3b–d), clearly suggest that CYP3A-expressing subpopulations of cells present in chemo-naive samples persist post-CTX.

To explore the clinical importance of divergent CYP3A coexpression patterns, we initially assessed whether high GATA6^hi^CYP3A^+^ or KRT17^hi^CYP3A^+^ protein expression was associated with participant outcome. This exploratory analysis demonstrated that coexpression of CYP3A, in the context of either GATA6 or KRT17, was significantly associated with poor overall survival for neoadjuvant mFFX but not GEM (Extended Data Fig. 6). Again, validation in suitably matched independent cohorts will be required to support these findings.

Assessment of GATA6/CYP3A/KRT17-expressing subpopulations in post-CTX samples (*n*_obs_ = 651,762 cells) revealed that mFFX treatment resulted in greater relative numbers of GATA6^+^CYP3A^+^KRT17^+^ and GATA6^+^ CYP3A^–^KRT17^+^ cells and fewer GATA6^+^CYP3A^–^KRT17^–^ cells than samples treated preoperatively with GEM (Fig. 6d). Dichotomization of post-CTX mFFX samples using GATA6 expression tertiles (high versus low) demonstrated that samples with low GATA6 expression (associated with longer survival) had increased numbers of GATA6^+^CYP3A^+^KRT17^+^ and GATA6^+^CYP3A^–^KRT17^–^ cells, whereas samples with high GATA6 expression (associated with shorter survival) had increased numbers of GATA6^+^CYP3A^–^KRT17^+^ and GATA6^–^CYP3A^+^KRT17^+^ cells but significantly fewer GATA6^+^CYP3A^–^KRT17^–^ cells (Fig. 6e). Taken together, these data suggest that CYP3A-expressing cell phenotypes may mediate resistance to mFFX neoadjuvant chemotherapy.

### CYP3A protein activity is associated with drug tolerance

**Fig. 7:**
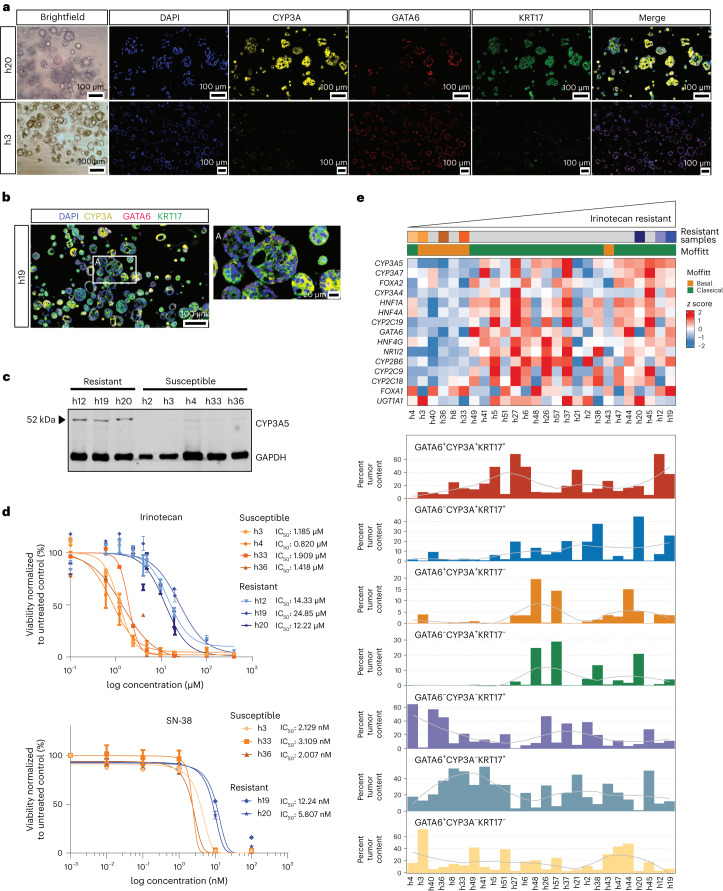
CYP3A protein expression is positively associated with irinotecan drug tolerance. **a**, Multiplexed IF of representative PDOs showing high relative CYP3A protein expression in resistant (h20) versus sensitive (h3) PDOs. **b**, Multiplexed IF of an irinotecan-resistant PDO (h19) showing mosaic GATA6/CYP3A/KRT17 protein expression. The ROI demarcated by a white box and labeled with i is shown at higher magnification. **c**, Western blot showing protein expression of CYP3A between PDOs exhibiting resistance or susceptibility to irinotecan. GAPDH is used as a loading control. **d**, Drug response curves showing half-maximal inhibitory concentration (IC_50_) values for both irinotecan and SN-38 in selected PDOs that are either relatively resistant or relatively susceptible. The results represent *n* = 3 independent biological experiments. Data are presented as mean values ± s.e.m. **e**, Relative enrichment of CYP3A^+^ ‘hybrid’ cell phenotypes in PDOs. Top, heat map showing mRNA expression of coexpressed genes associated with drug metabolism and phase I functionalization of compounds. Bottom, bar charts showing the percent tumor enrichment of GATA6/CYP3A/KRT17 cell populations as determined by multiplexed IF. PDOs in the top and bottom are identical and are ordered according to increasing irinotecan IC_50_ values. A LOESS regression line has been added to each bar plot. Source data

**Extended Data Fig. 8:**
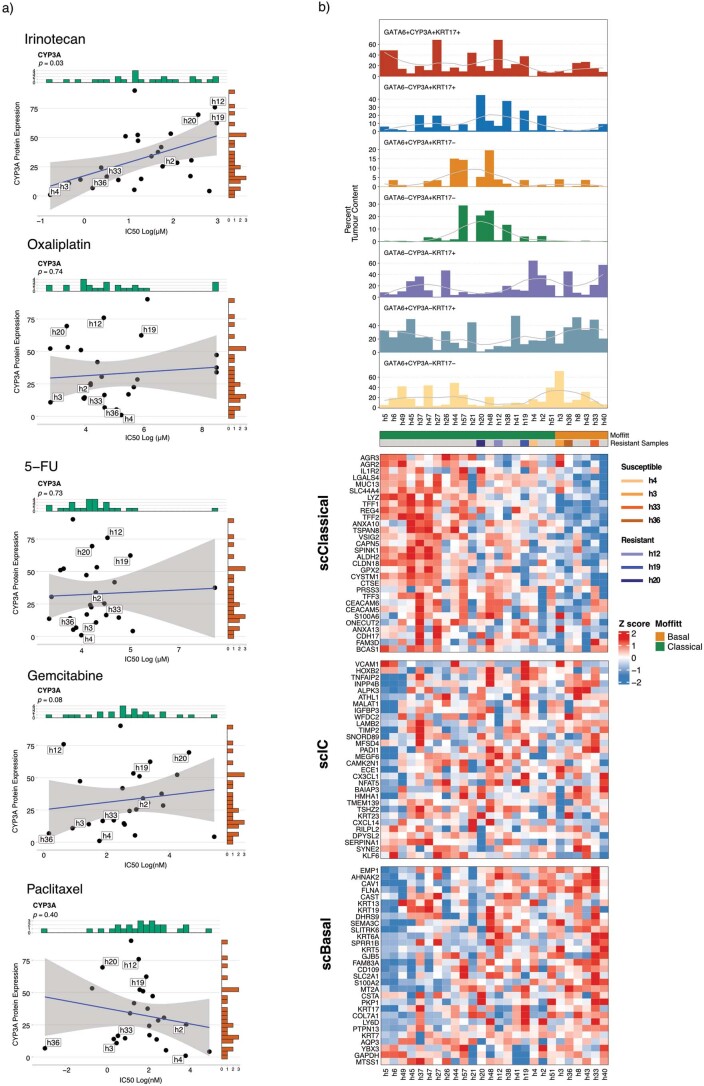
CYP3A protein expression in PDOs is positively correlated with irinotecan resistance. **a**) Scatter plots showing the correlations between CYP3A protein expression and Log (IC50) values for the indicated chemotherapies. Winsorized correlations (two-sided) were performed to reduce the effect of outliers. *P*-values are shown on each correlation plot with *P*-value ≤ 0.05 considered significant. The plots show a solid regression line and error bands representing 95% confidence intervals. *P*-values were not adjusted for multiple testing. **b**) CYP3A-positive ‘Hybrid’ cells overlap scClassical and scIC gene signatures. Top panel, bar plots showing the percent tumour content of GATA6/CYP3A/KRT17 hybrid cell types in PDOs. A LOESS regression line has been added to each bar plot. Bottom panel, heatmap showing the relative expression of gene signatures derived from single cell analyses that define distinct Classical (scClassical), Basal (scBasal) and Intermediate co-expressor (IC) cell phenotypes. PDOs are grouped by Moffitt subtype. Source data

To test whether CYP3A mediates chemotherapy resistance, we used a large panel of patient-derived organoids (PDOs) generated from chemo-naive, post-CTX and liver biopsy metastatic material (Extended Data Fig. 7c–g and Supplementary Tables 2 and 10). Thirty-one PDOs, representing 24 chemo-naive, 6 post-CTX and 1 liver biopsy, were characterized by RNA-seq (Supplementary Table 11), multiplexed IF (Supplementary Table 12) and therapeutic response to standard chemotherapies, including irinotecan, oxaliplatin, 5-fluorouracil, GEM and paclitaxel (Extended Data Fig. 7e). Transcriptomic analysis of PDAC-HD PDOs identified 25 PDOs exhibiting a classical subtype and 6 PDOs exhibiting a basal-like Moffitt subtype (Fig. 7e). Analysis using single-cell transcriptomic cell signatures^17^ identified PDOs exhibiting predominant scClassical and scBasal profiles, with the majority exhibiting scIC transcriptomic profiles (Extended Data Fig. 8b). Multiplexed IF for GATA6/KRT17/CYP3A revealed that PDOs, exhibiting scIC transcriptomic profiles, were enriched for GATA6, KRT17 and CYP3A coexpressing ‘hybrid’ cell phenotypes.

Consistent with previous studies, we observed heterogenous responses in PDOs to different chemotherapies (Extended Data Fig. 7e–g)^46^. Ranking of PDOs according to relative irinotecan sensitivity identified seven PDOs exhibiting either relative resistance (h12, h19 and h20) or sensitivity to irinotecan (h3, h4, h33 and h36). PDOs resistant to irinotecan exhibited predominant Moffitt classical transcriptomic profiles, whereas five of the six most susceptible PDOs exhibited Moffitt basal transcriptomic profiles (Fig. 7e). Importantly, CYP3A protein expression, as determined by western blotting and IF, was positively correlated with irinotecan resistance in PDOs (Fig. 7a–e and Extended Data Fig. 8). Similarly, genes involved in drug metabolism and phase I functionalization of compounds were enriched in irinotecan-resistant PDOs, suggesting that coordinated networks of drug-metabolizing genes mediate irinotecan resistance (Fig. 7e).

Multiplexed IF data demonstrated a relative increase in GATA6-, KRT17- and CYP3A-coexpressing ‘hybrid’ phenotypes in irinotecan-resistant PDOs (Fig. 7e). In particular, the percent organoid content of GATA6^+^KRT17^+^CYP3A^+^ and GATA6^–^KRT17^+^CYP3A^+^ cells was enriched in irinotecan-resistant PDOs. The enrichment of these ‘hybrid’ cell types was similarly observed in post-CTX samples treated preoperatively with mFFX (Fig. 6a).

**Fig. 8:**
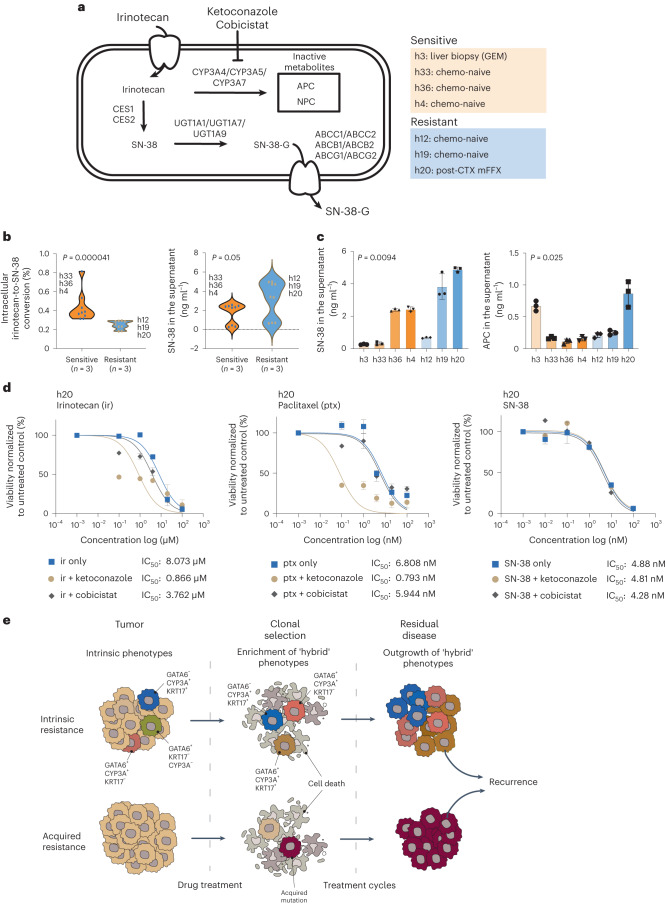
CYP3A activity mediates irinotecan tolerance in CYP3A^+^ PDOs. **a**, Irinotecan is converted to the active metabolite SN-38 in liver and small intestinal epithelial cells and also pancreatic cancer cells. CYP3A proteins may metabolize irinotecan into inactive metabolites APC and NPC, leading to drug tolerance. SN-38 may also undergo glucuronidation and be exported from cancer cells. CYP3A inhibitors, such as ketoconazole and cobicistat, may overcome irinotecan drug tolerance by increasing the accumulation of SN-38. **b**, Compound analysis by UPLC–MS/MS of irinotecan metabolites in relative resistant (*n* = 3) and relative susceptible (*n* = 3) PDOs showing intracellular irinotecan-to-SN-38 conversion (left) and SN-38 accumulation in the supernatant (right). Biological replicates (*n* = 3) for the representative PDOs are shown in the plots. Wilcoxon rank-sum test two-sided *P* values are shown on the plots. *P* values were not adjusted for multiple testing. **c**, Compound analysis by UPLC–MS/MS of relative resistant and relative susceptible PDOs showing the accumulation of SN-38 or inactive metabolite APC in the supernatant following irinotecan treatment. The results represent *n* = 3 independent biological experiments. Data are presented as mean ± s.d. One-way analysis of variance (two-tailed) *P* values are shown on the plots. *P* values were not adjusted for multiple testing. **d**, Treatment of an irinotecan-resistant PDO (h20) with irinotecan, paclitaxel and SN-38 in combination with either ketoconazole or cobicistat as indicated. Combination treatment with ketoconazole increases drug sensitivity to irinotecan and paclitaxel but not SN-38. The results represent *n* = 3 independent biological experiments. Data are presented as mean ± s.e.m. IC_50_ values are provided for the indicated treatments. **e**, Alternative models to explain drug tolerance and persistence in post-CTX samples. Intrinsic resistance: treatment-mediated selection of preexisting drug-tolerant phenotypes may shape residual disease. This process may involve non-genetic mechanisms, including intrinsic and/or drug-induced expression of drug-detoxifying genes and/or a transition toward basal-like or ‘hybrid’ states from predominant classical states due to intrinsic neoplastic plasticity. Acquired resistance: rare subclones acquire a drug-resistant driver alteration before or during therapy. These resistant clones expand and eventually drive relapse due the clonal acquisition of the preexisting drug-resistant mechanism. Source data

**Extended Data Fig. 9:**
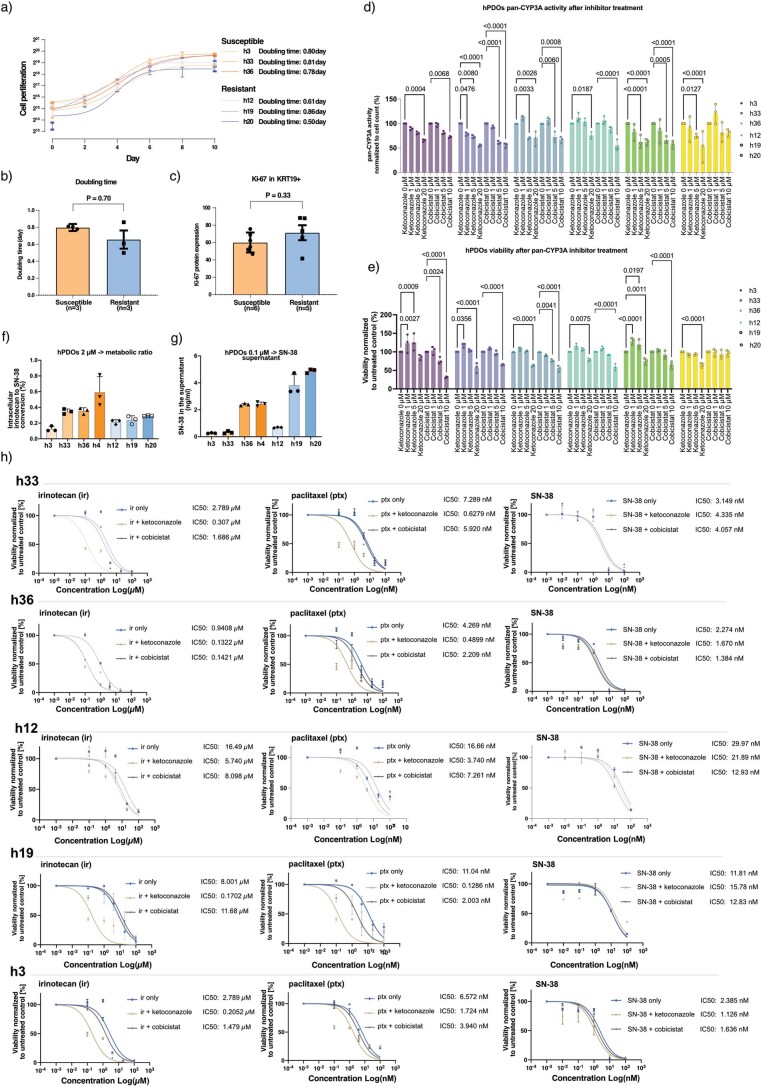
CYP3A activity mediates irinotecan tolerance in CYP3A-positive PDOs. **a**) Cell proliferation assay showing doubling time for resistant (n = 3) and susceptible (n = 3) PDOs. The results represent n = 3 independent biological experiments. **b**) Bar plot showing doubling time between resistant (n = 3) and susceptible (n = 3) PDOs. Mann-Whitney (two-sided) *P*-value is shown on the plot. *P*-value was not adjusted for multiple testing. Bar plots represent mean values ± s.d. **c**) Bar plot of Ki67 protein expression in selected resistant (n = 5) and susceptible (n = 6) PDOs. Mann-Whitney (two-sided) *P*-value is shown on the plots. *P*-value was not adjusted for multiple testing. Bar plots represent mean values ± s.d. **d**) CYP3A enzyme activity in selected PDOs as determined by luminescence assay. CYP3A enzyme activity was normalized to total cell number. PDOs were treated with increasing concentrations of the CYP3A inhibitors ketoconazole and cobicistat to determine the optimum concentrations for combination treatments. Dunnett’s multiple comparison test (two-sided) was performed to identify concentrations sufficient to significantly inhibit CYP3A activity. *P*-values adjusted by multiple correction are shown on the plot. Bar plots show mean values of n = 3 independent biological experiments ± s.d. **e**) Cell viability assays showing PDO responses to increasing concentrations of the CYP3A inhibitors ketoconazole and cobicistat. Dunnett’s multiple comparison test (two-sided) was performed to identify concentrations sufficient to significantly reduce cell viability. *P*-values adjusted by multiple correction are shown on the plot. Bar plots show mean values of n = 3 independent biological replicates ± s.d. **f**) Compound analysis by UPLC-MS/MS of relative resistant and relative susceptible PDOs showing relative irinotecan to SN-38 conversion. Bar plots show mean values of n = 3 independent biological experiments ± s.d. **g**) Compound analysis by UPLC-MS/MS of relative resistant and relative susceptible PDOs showing relative SN-38 in the supernatant. Bar plots show mean values of n = 3 independent biological experiments ± s.d. **h**) Treatment of selected PDOs with irinotecan, SN-38, or paclitaxel in combination with either ketoconazole or cobicistat as indicated. Combination treatment with ketoconazole increases drug sensitivity to irinotecan and paclitaxel but not SN-38. IC50 values are provided for the indicated treatments. The results represent n = 3 independent biological experiments. Data are presented as mean values +/− s.e.m. Source data

CYP3A has been shown to convert irinotecan into the inactive metabolites 7-ethyl-10-[4-*N*-(5-aminopentanoic acid)-1-piperidino]-carbonyloxycamptothecin (APC) and 7-ethyl-10-(4-amino-1-piperidino)-carbonyloxycamptothecin (NPC) in both liver and intestinal epithelial cells (Fig. 8a)^47^. To further validate our hypothesis that CYP3A-mediated activity is involved in irinotecan resistance, we assessed CYP3A activity in selected PDOs (Extended Data Fig. 9d) and performed validated ultraperformance liquid chromatography–tandem mass spectrometry (UPLC–MS/MS) quantifications to track the metabolism of irinotecan following exposure to non-cytotoxic concentrations (Fig. 8a).

MS analysis demonstrated that resistant and susceptible PDOs take up irinotecan. Exposure of PDOs to 2 µM irinotecan demonstrated that less than 1% of the intracellular irinotecan was converted to intracellular SN-38 (Extended Data Fig. 9f). Importantly, irinotecan-resistant PDOs showed significantly lower metabolic ratios (intracellular SN-38:intracellular irinotecan) than irinotecan-sensitive organoids, suggesting that the resistance phenotype involves lower conversion of irinotecan to SN-38 (Fig. 8b,c), effects that were independent of proliferation (Extended Data Fig. 9a–c). In addition, resistant PDOs tended to show higher SN-38 concentrations in the supernatant, suggesting that the active transport of SN-38 may also contribute to irinotecan resistance (Fig. 8b,c). This was especially true for the irinotecan-resistant PDO h20, which exhibited the highest concentration of SN-38 in the supernatant (Fig. 8b,c and Extended Data Fig. 9g).

The CYP3A-mediated metabolite APC was inconclusively quantifiable in the majority of PDOs; however, physiologically relevant amounts were recorded in the supernatant, suggesting that APC is actively transported from PDOs (Fig. 8c). PDO h20 exhibited quantifiable levels of APC in both cells and supernatant and had the highest supernatant concentration of APC for the PDOs tested. Notably, PDO h20 was derived from a participant who received neoadjuvant mFFX and was enriched for GATA6^–^CYP3A^+^KRT17^+^ and GATA6^–^CYP3A^+^KRT17^–^ cell phenotypes. PDO h3, which shows relative susceptibility to irinotecan and was derived from a metastatic liver recurrence after first-line GEM monotherapy, was the only other organoid to produce high supernatant concentrations of APC.

To establish a direct role for CYP3A in irinotecan drug resistance, we treated selected resistant and susceptible PDOs with optimal concentrations of irinotecan in combination with either ketoconazole or cobicistat, potent pan-CYP3A inhibitors (Fig. 8d and Extended Data Fig. 9d,e,h). Treatment of resistant PDOs with irinotecan in combination with ketoconazole significantly increased sensitivity to irinotecan. Importantly, the ability of ketoconazole to sensitize resistant cells to irinotecan was not observed when the active metabolite SN-38 was used as a substitute (Fig. 8d and Extended Data Fig. 9h). CYP3A activity has also been shown to mediate paclitaxel resistance in pancreatic cancer cell lines^37^. Consistent with these findings, ketaconzole-mediated inhibition of CYP3A increased sensitivity to paclitaxel in irinotecan-resistant PDOs. Together these findings implicate CYP3A as an important mediator of irinotecan drug resistance in PDAC.

## Discussion

Drug resistance to standardized chemotherapy remains a significant challenge for PDAC, with relapse occurring in most individuals^5,8,9^. It is currently unclear how drug-resistant clones emerge and evolve during therapy. Two major mechanisms have been proposed to explain drug resistance (Fig. 8e). The first of these supposes that drug resistance arises from rare subclones that acquire a drug-resistant driver alteration (genetic) during therapy. In response to therapy, these resistant clones expand and eventually drive relapse due to the clonal acquisition of the preexisting drug-resistant mechanism. The second model proposes that drug-tolerant cells, or ‘persisters’, initially emerge from a preexisting subpopulation of cells that do not harbor classical drug-resistant driver alterations (non-genetic). These cells survive initial drug treatments by an epigenetic and/or transcriptional adaption that allows a drug-tolerant, slow-cycling ‘persister’ state to emerge. Clinically, this persister state resembles minimal residual disease from which relapse can occur if treatment is discontinued or if persister cells acquire a drug-resistant driver alteration due to continued drug therapy^48^.

The evidence presented herein strongly suggests that preexisting subpopulations of GATA6-, KRT17- and CYP3A-coexpressing cells survive the initial rounds of mFFX treatment to emerge as a persistent population of drug-resistant cells. Recent evidence has revealed that CYP3A expression in pancreatic cancer cells may mediate resistance to paclitaxel and tyrosine kinase inhibitors^37^. Here, we extend these findings to demonstrate that CYP3A activity is a mediator of irinotecan resistance. Pharmacological inhibition of CYP3A activity using the pan-CYP3A inhibitor ketoconazole sensitized resistant PDOs to irinotecan but not the active metabolite SN-38, directly implicating CYP3A activity in irinotecan resistance. Strikingly, GATA6-, KRT17- and CYP3A-coexpressing ‘hybrid’ cells were enriched in resistant PDOs, further implicating these cell phenotypes in chemotherapy resistance.

Recent studies have demonstrated that classical and basal-like phenotypes exist as a gene expression continuum with a ‘hybrid’ or IC state acting as a plastic intermediate^17^. Modulation of cell state by the addition of stromal cues or chemotherapy has been shown to shift this continuum from a classical state toward an induced ‘hybrid’ and/or basal-like (mesenchymal) state. Accordingly, the enrichment of ‘hybrid’ and basal-like states at the expense of classical-like states observed in post-CTX samples may reflect this underlying plasticity. Understanding how resistant neoplastic cell populations emerge following chemotherapy will be critical for the development of effective first-line and adjuvant therapies. Future studies comparing matched before and after treatment samples should provide critical new insights into the genetic and non-genetic mechanisms underpinning therapy resistance.

The number of individuals pretreated with chemotherapy is increasing. Neoadjuvant chemotherapy may improve outcomes for individuals with locally advanced and borderline cancer, and studies in resectable individuals are ongoing^10–15^. Resected tumor material represents a unique opportunity to tailor adjuvant treatment to residual cancer cells. Development of effective treatments for the persister cells identified in this study is therefore of the utmost importance. As increased CYP3A expression may increase resistance to both irinotecan and nab-paclitaxel, alternative chemotherapy combinations without these drugs, such as GEM–capecitabine, might be considered. Moreover, the enrichment of suppressive macrophage signatures and/or specific immunoregulators in neoadjuvant samples suggests that combination approaches targeting myeloid cells^49,50^ and/or major inhibitory checkpoint molecules^51^ may also enhance benefit.

## Methods

### Participant characteristics

The study was approved by the Ethics Committee of Heidelberg University for use of pancreatic cancer tissue (project numbers S-018/2020, S-708/2019 and S-083/2021). Participants were selected according to the following criteria: (1) individuals with locally advanced borderline unresectable PDAC (without metastases) who received surgical resection after mFFX or GEM-based therapy without any chemoradiation neoadjuvant therapy and (2) individuals with resectable PDAC at presentation who had upfront surgical resection without prior chemotherapy and/or chemoradiation (adjuvant chemo-naive). Participants after resection could receive adjuvant chemotherapy, but those who had chemoradiation were again excluded. Consecutive pseudoanonymized participants with prior consent and with usable samples of sufficient quality for investigation were identified based on the eligibility criteria. Additional pseudoanonymized IDs were then ascribed to each participant for experimental investigation, so blinded to all of the investigators and investigations. Clinical correlates were only ascribed once the experimental procedure results were obtained.

Cryopreserved and FFPE tissues were processed as described previously^52^. Hematoxylin and eosin (H&E) staining of cryopreserved and FFPE tissue was examined by a specialist pancreas pathologist (F.B.) for staging^53^ and tumor content in the Department of Pathology. RNA-seq was performed on cryopreserved PDAC tissues, and multiplexed IF was performed on PDAC FFPE tissues.

### Organoid generation and propagation

Human PDOs were cultivated as previously described by Tuveson and colleagues^54^ After being minced into pieces, the tumor tissue was digested using collagenase XI (Sigma)-containing digestion medium in a rotating shaker at 37 °C for 45 min. The dissociated cells were embedded in growth factor-reduced Matrigel and cultured in complete medium (advanced DMEM/F12 medium supplemented with HEPES (1×, Gibco), GlutaMAX (1×, Gibco), B-27 (1×, Gibco), Primocin (1 mg ml^–1^, InvivoGen), *N*-acetyl-l-cysteine (1 mM, Sigma), WNT3A-conditioned medium (50% (vol/vol)), RSPO1-conditioned medium (10% (vol/vol)), human epidermal growth factor (50 ng ml^–1^, Peprotech), gastrin I (10 nM, Tocris), human fibroblast growth factor 10 (100 ng ml^–1^, Prepotech), nicotinamide (10 mM, Sigma) and A83-01 (0.5 μM, Tocris)). Medium was changed twice a week. For continued passaging, organoids were recovered from the Matrigel using cold Cell Recovery Solution (Corning), further dissociated into single cells using TrypLE (Gibco) and embedded with fresh Matrigel. Organoid cell lines were checked for *KRAS* mutations by DNA Sanger sequencing.

### Sanger sequencing

DNA was extracted from snap-frozen human PDO cell pellets by the Sample Processing Lab using an AllPrep kit (Qiagen). Primers sequences for amplification and sequencing of exons of the *KRAS* gene that contain the G12/13 codons are listed below:

*KRAS* G12/13 forward: 5′-CTGGTGGAGTATTTGATAGTG-3′

*KRAS* G12/13 reverse: 5′-CTGTATCAAAGAATGGTCCTG-3′.

The following PCR conditions were used as previously described^54^ and specifically noted in that paper: ‘94 °C for 2 min; three cycles of 94 °C for 30 s, 64 °C for 30 s and 72 °C for 30 s; three cycles of 94 °C for 30 s, 61 °C for 30 s and 72 °C for 30 s; three cycles of 94 °C for 30 s, 58 °C for 30 s and 72 °C for 30 s and three cycles of 94 °C for 30 s, 57 °C for 30 s and 72 °C for 30 s, followed by 72 °C for 5 min and a hold at 4 °C’. PCR products were purified using a QIAquick PCR purification kit and sent to and sequenced by Eurofins. The resulting sequences were analyzed using Mutation Surveyor software (SoftGenetics).

### Pharmacological assay of organoids

Organoids were dissociated into single cells before being plated in 10 μl of Matrigel with 1,000 cells per well into white 96-well plates (Greiner). Chemotherapeutics were tested in triplicate: 5-fluorouracil (Sigma), irinotecan (Sigma) and oxaliplatin (Selleckchem) with concentrations ranging from 1.0 × 10^−7^ to 1.0 × 10^−3^ mol liter^–1^, GEM (Sigma), paclitaxel (Selleckchem) and SN-38 (Sigma) with concentrations ranging from 1.0 × 10^−10^ to 1.0 × 10^−6^ mol liter^–1^ and ketoconazole (Sigma) and cobicistat (MCE) ranging from 1.0 × 10^−6^ to 2.0 × 10^−5^ mol liter^–1^. In combination treatment, 5.0 × 10^−6^ mol liter^–1^ ketoconazole or cobicistat was combined with a corresponding dose of irinotecan, paclitaxel or SN-38. Reagents were dissolved in DMSO and added 72 h after plating. All treatment wells were normalized to 0.25% DMSO. After 96 h of treatment, cell viability was assessed using the CellTiter-Glo 3D cell viability assay (Promega), as per the manufacturer’s instructions, on a FLUOstar plate reader (BMG Labtech). Therapeutic results (viability versus dose) were analyzed with GraphPad software. A four-parameter log-logistic function with an upper limit equal to the mean of the DMSO values was fit to the drug response curve and IC_50_ value calculated.

### CYP3A activity assay in organoids

After 96 h of culture with or without CYP3A inhibitor, the pan-CYP3A activity of organoids was measured using a P450-Glo kit (Promega), as per the manufacturer’s instructions, on a FLUOstar plate reader (BMG Labtech). All activity measurements from each well were normalized to cell numbers using the CellTiter-Glo assay mentioned above.

### Histology for organoids

Organoids were fixed in 4% paraformaldehyde solution and embedded in paraffin. Sections were subjected to H&E and IF staining. The following primary antibodies were used for IF staining: KRT19 (ab7755, Abcam; 1:100), CYP3A5 (ab108624, Abcam; 1:200), KRT17 (sc393002, Santa Cruz; 1:50), GATA6 (AF1700, R&D; 1:100), Ki-67 (ab16667, Abcam; 1:200) and DAPI (D9542, Sigma; 1:1,000). Images of H&E and IF staining were acquired using imaging system Tissue-FAXS software (TissueGnostics). H&E images were acquired using a ×20 objective lens in brightfield. IF images were acquired using a ×20 objective lens with light-emitting diodes (LEDs) with specific light filters. IF images of negative-control sections were used to set the appropriate gating to exclude background IF and non-specific binding signals. The expression level of each protein was calculated by the percentage of protein-positive-stained cells in DAPI-positive cells.

### Western blotting

Protein samples from organoids were lysed in RIPA lysis buffer^55^ with protease inhibitor cocktail (Sigma) and phosphatase inhibitor (Sigma) and quantified using a Pierce BCA protein assay kit (Thermo Fisher). Following SDS–PAGE and transfer to PVDF membranes (Bio-Rad, 1704273), membranes were blocked in Tris-buffered saline containing 5% bovine serum albumin (BSA) and 0.1% Tween 20 (TBS-T) for 1 h before incubation with primary antibody (CYP3A5, ab108624, Abcam, 1:1,000; GAPDH, cs2118, Cell Signaling, 1:1,000) overnight at 4 °C. After washing three times in TBS-T and incubating with species-corresponding secondary antibodies (anti-mouse IgG, LI-COR, 1:10,000; anti-rabbit IgG, LI-COR, 1:10,000), membranes were visualized with an ODYSSEY CLx (LI-COR) imaging system.

### Compound analysis by UPLC–MS/MS

Organoids were treated for 96 h with 0.1 µM or 2 µM irinotecan (<IC_50_) to prevent selection bias from killing most of the organoid population. Non-lethal drug concentrations were chosen to allow metabolic phenotyping of intracellular and extracellular (supernatant) concentrations of irinotecan, SN-38, APC and NPC.

Intracellular and supernatant concentrations were quantified with a validated UPLC–MS/MS assay following the guidelines of the European Medicines Agency (EMA) and US Food and Drug Administration (FDA) and on bioanalytical method validation (http://www.ema.europa.eu/docs/en_GB/document_library/Scientific_guideline/2011/08/WC500109686.pdf and http://www.fda.gov/downloads/Drugs/GuidanceComplianceRegulatoryInformation/Guidances/ucm070107.pdf). Each of four performed validation runs included blank and internal standard controls, seven calibration samples (twofold) and four quality control (QC) concentrations (sixfold). The assays fully complied with the applicable parts of the recommendations of the US FDA and EMA.

Optimized MS/MS parameters for the detection of irinotecan and its metabolites can be found in Supplementary Table 3. The calibrated range was 10–10,000 pg ml^–1^, showing linear regression coefficients of >0.99. Overall accuracies (interday and intraday) were between 90.0 and 114.0% with a corresponding precision of <15%. A Xevo-TQ-S tandem mass spectrometer (Waters) coupled to an Acquity classic UPLC (Waters) and equipped with a heated electrospray ionization source was used for quantification. Determinations were performed with selected reaction monitoring using collision-induced dissociation with argon in the positive ion mode. Chromatographic separation was performed on a BEH C18 column (50 × 2.1 mm and 1.7 µm, Waters) with a linear gradient from 5 to 75% acetonitrile (ACN) + 0.1% formic acid in 1.2 min (corresponding decrease of aqueous eluent: 19:1 water:ACN + 0.1% formic acid) at a flow rate of 0.5 ml min^–1^.

Organoids were lysed using 300 µl of 5% aqueous NH_3_, from which 100 µl was withdrawn for analysis (study samples). Calibration and QC samples were prepared by spiking 25 µl of calibration or QC spike solution (corresponding sample concentration calibration: 10, 30, 100, 300, 1,000, 3,000 and 10,000 pg ml^–1^; QC: 10, 30, 3,750 and 7,500 pg ml^–1^) into 100 µl of cell lysate. All samples (lysed organoids or supernatants) were spiked with 25 µl of internal standard solution (irinotecan-D_6_ and SN-38-D_6_), and study samples were additionally spiked with 25 µl of blank solution for volume compensation. Irinotecan and metabolites were extracted with protein precipitation using 300 µl of acetonitrile containing 0.1% formic acid. After shaking and centrifugation at 16,100*g* for 5 min, extracts were transferred to 96-well collection plates and evaporated with a blowdown evaporator (Ultravap, Porvair Sciences). After addition of 100 µl of a mixture of water:ACN (3:1 (vol/vol)) containing 0.1% formic acid, 20 µl was injected into the UPLC–MS/MS system for analysis.

Measured drug concentrations in cellular lysates were normalized to protein content of the sample, which were evaluated using a commercial BCA assay kit (Pierce BCA Protein Assay kit, Thermo Scientific). Measurements were performed as previously described^56^. Specifically, as noted in that paper, a calibration curve with nine standard sample concentrations of BSA (0–2,000 µg ml^–1^) was prepared. Wells of a 96-well plate were loaded with 8 µl of BSA standard or the respective sample and 64 µl of the working solution (a mixture of reagent A and reagent B contained in the kit). After 30 min of light-protected incubation at 37 °C, absorption was read at 562 nm using a Spectramax plate reader (Molecular Devices), and protein content was calculated.

### Tissue processing and next-generation sequencing

LCM was performed on cryopreserved tissue samples from individuals after resection and before any adjuvant therapy, according to previously described methods^29,57^. RNA from bulk and LCM tumor specimens was isolated using an AllPrep DNA/RNA/miRNA Universal kit (Qiagen). RNA samples with an RNA integrity number of ≥8, a 28S/18S ratio of ≥1.0 and a DV200 of >70% were considered suitable for high-throughput sequencing. High-throughput sequencing library preparation was performed with an Illumina TruSeq stranded mRNA kit (Illumina, 20020595) with IDT unique dual indices (Illumina, 20022371) following the manufacturer’s recommendations. Five hundred nanograms of total RNA was used as input. For each library, at least 57 million mapped reads were produced for downstream analysis.

### IF assays

IF staining was accomplished using 4-µm-thin FFPE tissue sections, as described earlier^58^. Whole sections were captured using a TissueGnostics Fluorescence Imaging System (TissueGnostics), with a fluorescence microscope unit (Observer. Z1, Zeiss) and a Lumencor Sola SE III 365- to 730-nm LED light source (AHF Analysentechnik). Captured images were analyzed using StrataQuest software version 7.0 (TissueGnostics), which calculated the intensity of the fluorescence signals in each single cell within individual tissue sections. Cells containing high-intensity target signals were selected by partitioning cell fluorescent scattergrams against DAPI or other referenced co-stained target signals into upper expression quantiles. The expression level of each marker protein was calculated as the percentage of stained cells relative to DAPI and/or stated reference protein. Biomarker protein expression in individual cells was gated into high, medium or low expression tertiles, and cell-specific expression was quantified for each sample. The antibodies used in this study are listed in Supplementary Tables 13 and 14.

### RNA-seq analysis

RNA-seq data were aligned using STAR version 2.5.3a via a DKFZ internal next-generation sequencing data-processing pipeline^59^. Downstream transcriptomic analysis was performed as previously described^24,26–28^. Briefly, merged count data obtained from the DKFZ processing pipeline and representing chemo-naive and post-CTX samples were batch corrected using the sva R package. Batch-corrected count data were subsequently logR transformed using the DESeq2 R package to generate normalized gene expression values. The logR-normalized data were used for all downstream analyses unless otherwise specified.

### Subtyping analysis

Subtyping analysis was performed on samples using gene expression signatures representing Moffitt^24,46^, Collisson^26^, Bailey^27^ and Notta^28^ subtypes. To classify samples according to subtype, logR-normalized gene expression values were clustered using the ConsensusClusterPlus R package. Samples were subsequently assigned to a specific subtype based on the results of the clustering analysis. Heat maps representing subtype clusters and showing representative subtype-specific genes were generated using the ComplexHeatmap R package. The PurIST algorithm was used as an orthogonal measure of basal-like subtype status in samples and was performed using normalized gene expression data, as previously described^25^. PurIST scores approaching 1 indicate an increased likelihood that the sample is basal like.

Gene expression signatures representing Bailey GPs, single-cell states or malignant lineage or state programs were obtained from Bailey et al.^27^, Raghavan et al.^17^ and Hwang et al.^16^, respectively. These gene expression signatures were used to cluster samples and/or generate gene set enrichment scores. Gene set enrichment scores were generated using the GSVA R package. t-SNE analysis of gene expression or gene set enrichment scores was performed using the Rtsne R package. Volcano plots were generated using the ggplot2 R package. These plots represent the set of differentially expressed genes between post-CTX samples treated with either GEM or mFFX. Hwang et al.^16^ malignant lineage and state program genes significantly enriched in the indicted sample group (log_2_ (fold change) > 1 and –log_10_ (adjusted *P* value) > 2) were highlighted in relevant plots using the gghighlight R package. The ggstatsplot R package was used to assess the significance of single-cell subtype signature enrichment.

### WGCNA

WGCNA, as implemented by the WGCNA R package, was performed using logR-normalized gene expression data. WGCNA was performed as previously described^27^. Specifically, ‘WGCNA clusters genes into network GPs using a topological overlap measure (TOM) that represents a highly robust measure of network interconnectedness and provides a measure of connection strength between two adjacent genes and all other genes in a network. Genes were clustered using 1 – TOM as the distance measure and GPs defined as branches of the resulting cluster tree using a dynamic branch-cutting algorithm. Module eigengene values were used as a measure of GP expression in each sample.’ Module eigengene values were used to identify GPs significantly enriched in either chemo-naive or post-CTX samples. Pathway enrichment analysis (see Supplementary Table 7 for the list of genes representing each GP) was performed using the clusterProfiler and ReactomePA R packages. Networks representing GPs (Fig. 4b) were generated using the Reactome FI Cytoscape plugin 8.05 (https://apps.cytoscape.org/apps/reactomefiplugin) in Cytoscape version 3.9.1 (https://cytoscape.org)^60^.

### Stromal cell enrichment analysis

Stromal cell type and/or phenotype enrichment in samples was estimate using xCell^61^, as implemented by the immunedeconv R package (Fig. 2). The logR-normalized gene expression values were used to obtain xCell enrichment scores. Enrichment scores for gene signatures representing Grünwald subTMEs^20^, Öhlund CAF phenotypes^62^ or Hwang et al.^16^ fibroblast programs were generated using the GSVA R package. Volcano plots were generated as described above using the set of differentially expressed genes between post-CTX samples treated with either GEM or mFFX. Immunomodulatory gene expression values were compared between samples as indicated. Correlations between immunomodulatory factors were generated and visualized using the corrplot R package.

### Multiplexed IF cell-type analysis

GATA6, KRT17 and CYP3A cell counts obtained from StrataQuest image processing, as described above, were used for enrichment analysis. Bar and pie statistical plots were generated from individual cell counts using the ggstatsplot R package. Ternary plots were generated using the ggtern R package^63^. The relative percent enrichment of GATA6, KRT17 and/or CYP3A protein expression was calculated by dividing individual cell-type counts by the sum of all cell-type counts in each sample and multiplying by 100. Bar plots representing percent tumor cell enrichment were generated using the ggpubr R package.

### Reanalysis of scRNA-seq data

Single-cell RNA-seq data published in Hwang et al.^16^ was reanalyzed using the scanpy Python package, version 1.9.3 (https://scanpy.readthedocs.io/en/stable/api.html)^64^. UMAP embeddings and dot plots were generated using well-annotated scanpy functions.

### Survival analysis

Survival was estimated using the Kaplan–Meier method, as implemented by the survminer R package. Participants were stratified by transcriptomic subtype or protein expression values as indicated. Forest plots were generated using the ggforestplot R package.

### Statistics and reproducibility

No statistical methods were used to predetermine sample sizes, but our sample sizes are similar to those published in previous publications^16,17,24,26,27^. Participant selection was performed blind to clinical variables. Data collection and analysis were not performed blind to the conditions of the experiment. Informed consent was obtained from the participants of this study. Research findings do not apply to one sex or gender only. The gender of each participant was collected by consent and was self-reported. Information on gender is provided in Supplementary Tables 1, 2, 4 and 5. For tissue-based findings, 171 unique participants were included in the study, with 100 males and 71 females taking part. For RNA-seq, 56 males and 41 females were included in the analysis (Supplementary Tables 1 and 4). For multiplexed IF (Supplementary Tables 1 and 5), 71 males and 51 females were included in the analysis. For PDOs, organoids derived from 11 males and 20 females were included in the analysis (Supplementary Table 10). No gender-based analyses are shown, and no significant associations with gender were observed in the data. All source data comprise a participant identifier that can be used to disaggregate the data based on gender. All experiments are representative of at least three independent biological experiments. H&E and IF images for samples or PDOs are representative of at least three independent IF experiments on the same region of interest or PDO. No data points were excluded from the analyses. Data distributions were assumed to be normal, but this was not formally tested. *P* values of ≤0.05 were considered significant. Further information on research design is available in the Nature Research Reporting Summary linked to this article.

### Reporting summary

Further information on research design is available in the Nature Portfolio Reporting Summary linked to this article.

### Supplementary information

Reporting Summary

Supplementary Tables Supplementary Tables 1–14. Clinical and processed data representing the minimum dataset.

### Source data

Source Data Fig. 1 Statistical source data.

Source Data Fig. 2 Statistical source data.

Source Data Fig. 3 Statistical source data.

Source Data Fig. 4 Statistical source data.

Source Data Fig. 5 Statistical source data.

Source Data Fig. 6 Statistical source data.

Source Data Fig. 7 Statistical source data.

Source Data Fig. 8 Statistical source data.

Source Data Extended Data Fig. 1 Statistical source data.

Source Data Extended Data Fig. 2 Statistical source data.

Source Data Extended Data Fig. 3 Statistical source data.

Source Data Extended Data Fig. 4 Statistical source data.

Source Data Extended Data Fig. 5 Statistical source data.

Source Data Extended Data Fig. 6 Statistical source data.

Source Data Extended Data Fig. 7 Statistical source data.

Source Data Extended Data Fig. 8 Statistical source data.

Source Data Extended Data Fig. 9 Statistical source data.

Source Data Fig. 7c Uncropped blot

## Data Availability

All data relevant to this study are available from the corresponding authors upon request. All processed data, including normalized expression data for participant samples and PDOs, multiplexed IF and experimental results, are provided in the Supplementary Tables. RNA-seq data are available at the European Genome–Phenome Archive under accession number EGAS00001007143. RNA-seq data published in Hwang et al.^16^ and Brunton et al.^31^ were obtained from Gene Expression Omnibus accession number GSE202051 and BioProject accession number PRJNA630992, respectively. Source data are provided with this paper.

## Code availability

No custom algorithms were used in this study. Analyses used existing code made available through referenced software packages. Code used in this study is available upon request.

## References

[1] Siegel RL, Miller KD, Fuchs HE, Jemal A (2022). Cancer statistics, 2022. CA Cancer J. Clin..

[2] Cunningham D (2009). Phase III randomized comparison of gemcitabine versus gemcitabine plus capecitabine in patients with advanced pancreatic cancer. J. Clin. Oncol..

[3] Von Hoff DD (2013). Increased survival in pancreatic cancer with nab-paclitaxel plus gemcitabine. N. Engl. J. Med..

[4] Conroy T (2011). FOLFIRINOX versus gemcitabine for metastatic pancreatic cancer. N. Engl. J. Med..

[5] Neoptolemos JP (2004). A randomized trial of chemoradiotherapy and chemotherapy after resection of pancreatic cancer. N. Engl. J. Med..

[6] Neoptolemos JP (2010). Adjuvant chemotherapy with fluorouracil plus folinic acid vs gemcitabine following pancreatic cancer resection: a randomized controlled trial. JAMA.

[7] Neoptolemos JP (2017). Comparison of adjuvant gemcitabine and capecitabine with gemcitabine monotherapy in patients with resected pancreatic cancer (ESPAC-4): a multicentre, open-label, randomised, phase 3 trial. Lancet.

[8] Conroy T (2018). FOLFIRINOX or gemcitabine as adjuvant therapy for pancreatic cancer. N. Engl. J. Med..

[9] Xu Z (2021). Clinical impact of molecular subtyping of pancreatic cancer. Front. Cell Dev. Biol..

[10] Ghaneh P (2020). ESPAC-5F: four-arm, prospective, multicenter, international randomized phase II trial of immediate surgery compared with neoadjuvant gemcitabine plus capecitabine (GEMCAP) or FOLFIRINOX or chemoradiotherapy (CRT) in patients with borderline resectable pancreatic cancer. J. Clin. Oncol..

[11] Katz MHG (2021). Alliance A021501: preoperative mFOLFIRINOX or mFOLFIRINOX plus hypofractionated radiation therapy (RT) for borderline resectable (BR) adenocarcinoma of the pancreas. J. Clin. Oncol..

[12] Versteijne E (2022). Neoadjuvant chemoradiotherapy versus upfront surgery for resectable and borderline resectable pancreatic cancer: long-term results of the Dutch randomized PREOPANC trial. J. Clin. Oncol..

[13] Hackert T (2016). Locally advanced pancreatic cancer: neoadjuvant therapy with folfirinox results in resectability in 60% of the patients. Ann. Surg..

[14] Maeda S (2022). Pathological treatment response has different prognostic implications for pancreatic cancer patients treated with neoadjuvant chemotherapy or chemoradiotherapy. Surgery.

[15] Wang-Gillam A (2016). Nanoliposomal irinotecan with fluorouracil and folinic acid in metastatic pancreatic cancer after previous gemcitabine-based therapy (NAPOLI-1): a global, randomised, open-label, phase 3 trial. Lancet.

[16] Hwang WL (2022). Single-nucleus and spatial transcriptome profiling of pancreatic cancer identifies multicellular dynamics associated with neoadjuvant treatment. Nat. Genet..

[17] Raghavan S (2021). Microenvironment drives cell state, plasticity, and drug response in pancreatic cancer. Cell.

[18] Tempero MA (2021). Pancreatic adenocarcinoma, version 2.2021, NCCN clinical practice guidelines in oncology. J. Natl Compr. Canc. Netw..

[19] Amin, M. B. et al. (eds) AJCC Cancer Staging Manual 8th edn (Springer, 2017).

[20] Grünwald BT (2021). Spatially confined sub-tumor microenvironments in pancreatic cancer. Cell.

[21] Michelakos T (2021). Tumor microenvironment immune response in pancreatic ductal adenocarcinoma patients treated with neoadjuvant therapy. J. Natl Cancer Inst..

[22] Mota Reyes C (2020). Neoadjuvant therapy remodels the pancreatic cancer microenvironment via depletion of protumorigenic immune cells. Clin. Cancer Res..

[23] Peng H (2021). Neoadjuvant FOLFIRINOX therapy is associated with increased effector T cells and reduced suppressor cells in patients with pancreatic cancer. Clin. Cancer Res..

[24] Moffitt RA (2015). Virtual microdissection identifies distinct tumor- and stroma-specific subtypes of pancreatic ductal adenocarcinoma. Nat. Genet..

[25] Rashid NU (2020). Purity independent subtyping of tumors (PurIST), a clinically robust, single-sample classifier for tumor subtyping in pancreatic cancer. Clin. Cancer Res..

[26] Collisson EA (2011). Subtypes of pancreatic ductal adenocarcinoma and their differing responses to therapy. Nat. Med..

[27] Bailey P (2016). Genomic analyses identify molecular subtypes of pancreatic cancer. Nature.

[28] Chan-Seng-Yue M (2020). Transcription phenotypes of pancreatic cancer are driven by genomic events during tumor evolution. Nat. Genet..

[29] Aung KL (2018). Genomics-driven precision medicine for advanced pancreatic cancer: early results from the COMPASS trial. Clin. Cancer Res..

[30] O’Kane GM (2020). GATA6 expression distinguishes classical and basal-like subtypes in advanced pancreatic cancer. Clin. Cancer Res..

[31] Brunton H (2020). *HNF4A* and *GATA6* loss reveals therapeutically actionable subtypes in pancreatic cancer. Cell Rep..

[32] Kloesch, B. et al. A GATA6-centred gene regulatory network involving HNFs and ΔNp63 controls plasticity and immune escape in pancreatic cancer. *Gut* **71**, 766–777 (2021). 10.1136/gutjnl-2020-321397 PMC9733634 33846140

[33] Martinelli P (2017). GATA6 regulates EMT and tumour dissemination, and is a marker of response to adjuvant chemotherapy in pancreatic cancer. Gut.

[34] Kalisz M (2020). HNF1A recruits KDM6A to activate differentiated acinar cell programs that suppress pancreatic cancer. EMBO J..

[35] O’Kane GM (2020). GATA6 expression distinguishes classical and basal-like subtypes in advanced pancreatic cancer. Clin. Cancer Res..

[36] Roa-Peña L (2019). Keratin 17 identifies the most lethal molecular subtype of pancreatic cancer. Sci. Rep..

[37] Noll EM (2016). CYP3A5 mediates basal and acquired therapy resistance in different subtypes of pancreatic ductal adenocarcinoma. Nat. Med..

[38] Ding X, Kaminsky LS (2003). Human extrahepatic cytochromes P450: function in xenobiotic metabolism and tissue-selective chemical toxicity in the respiratory and gastrointestinal tracts. Annu. Rev. Pharmacol. Toxicol..

[39] Krishna DR, Klotz U (1994). Extrahepatic metabolism of drugs in humans. Clin. Pharmacokinet..

[40] Pavek P, Dvorak Z (2008). Xenobiotic-induced transcriptional regulation of xenobiotic metabolizing enzymes of the cytochrome P450 superfamily in human extrahepatic tissues. Curr. Drug Metab..

[41] Hwang-Verslues WW, Sladek FM (2010). HNF4α—role in drug metabolism and potential drug target?. Curr. Opin. Pharmacol..

[42] Buck E (2019). Tumor response to irinotecan is associated with CYP3A5 expression in colorectal cancer. Oncol. Lett..

[43] Greenhalf W (2014). Pancreatic cancer hENT1 expression and survival from gemcitabine in patients from the ESPAC-3 trial. J. Natl Cancer Inst..

[44] Aughton K (2021). hENT1 predicts benefit from gemcitabine in pancreatic cancer but only with low *CDA* mRNA. Cancers.

[45] Okamura, Y. et al. Concordance of human equilibrative nucleoside transporter-1 expressions between murine (10D7G2) and rabbit (SP120) antibodies and association with clinical outcomes of adjuvant chemotherapy for pancreatic cancer: a collaborative study from the JASPAC 01 trial. *Cancer Rep.* **5**, e1507 (2021). 10.1002/cnr2.1507 PMC9124504 34327872

[46] Tiriac H (2018). Organoid profiling identifies common responders to chemotherapy in pancreatic cancer. Cancer Discov..

[47] Santos A (2000). Metabolism of irinotecan (CPT-11) by CYP3A4 and CYP3A5 in humans. Clin. Cancer Res..

[48] Bailey P (2023). Refining the treatment of pancreatic cancer from big data to improved individual survival. Function.

[49] Candido JB (2018). CSF1R^+^ macrophages sustain pancreatic tumor growth through T cell suppression and maintenance of key gene programs that define the squamous subtype. Cell Rep..

[50] Steele CW (2016). CXCR2 inhibition profoundly suppresses metastases and augments immunotherapy in pancreatic ductal adenocarcinoma. Cancer Cell.

[51] Werba G (2023). Single-cell RNA sequencing reveals the effects of chemotherapy on human pancreatic adenocarcinoma and its tumor microenvironment. Nat. Commun..

[52] Steele NG (2020). Multimodal mapping of the tumor and peripheral blood immune landscape in human pancreatic cancer. Nat. Cancer.

[53] Brierley, J. D., Gospodarowicz, M. K. & Wittekind, C. *TNM Classification of Malignant Tumours* (John Wiley & Sons, 2017).

[54] Boj SF (2015). Organoid models of human and mouse ductal pancreatic cancer. Cell.

[55] Janes KA (2015). An analysis of critical factors for quantitative immunoblotting. Sci. Signal..

[56] Nilles J, Weiss J, Theile D (2022). Crystal violet staining is a reliable alternative to bicinchoninic acid assay-based normalization. Biotechniques.

[57] Roessler S (2015). Integrative genomic and tanscriptomic characterization of matched primary and metastatic liver and colorectal carcinoma. Int. J. Biol. Sci..

[58] Xie L (2019). Effects of neoadjuvant FOLFIRINOX and gemcitabine-based chemotherapy on cancer cell survival and death in patients with pancreatic ductal adenocarcinoma. Oncotarget.

[59] Reisinger E (2017). OTP: an automatized system for managing and processing NGS data. J. Biotechnol..

[60] Shannon P (2003). Cytoscape: a software environment for integrated models of biomolecular interaction networks. Genome Res..

[61] Aran D, Hu Z, Butte AJ (2017). xCell: digitally portraying the tissue cellular heterogeneity landscape. Genome Biol..

[62] Öhlund D (2017). Distinct populations of inflammatory fibroblasts and myofibroblasts in pancreatic cancer. J. Exp. Med..

[63] Hamilton NE, Ferry M (2018). ggtern: ternary diagrams using ggplot2. J. Stat. Softw..

[64] Wolf FA, Angerer P, Theis FJ (2018). SCANPY: large-scale single-cell gene expression data analysis. Genome Biol..

