# Supplementary material for: Persister cell phenotypes contribute to poor patient outcomes after neoadjuvant chemotherapy in PDAC
Source: Nat Cancer. 2023 Sep 7;4(9):1362–81. doi: 10.1038/s43018-023-00628-6 (PMC10518256; doi:10.1038/s43018-023-00628-6)
Supplement: Supplementary file 1 — Reporting Summary [file 43018_2023_628_MOESM1_ESM.pdf]

nature portfolio

Corresponding author(s): Peter Bailey

Last updated by author(s): Jun 19, 2023

Reporting Summary

Nature Portfolio wishes to improve the reproducibility of the work that we publish. This form provides structure for consistency and transparency in reporting. For further information on Nature Portfolio policies, see our Editorial Policies and the Editorial Policy Checklist.

Statistics

For all statistical analyses, confirm that the following items are present in the figure legend, table legend, main text, or Methods section.

n/a	Confirmed	
☐	☒	The exact sample size (*n*) for each experimental group/condition, given as a discrete number and unit of measurement
☐	☒	A statement on whether measurements were taken from distinct samples or whether the same sample was measured repeatedly
☐	☒	The statistical test(s) used AND whether they are one- or two-sided<br>*Only common tests should be described solely by name; describe more complex techniques in the Methods section.*
☐	☒	A description of all covariates tested
☐	☒	A description of any assumptions or corrections, such as tests of normality and adjustment for multiple comparisons
☐	☒	A full description of the statistical parameters including central tendency (e.g. means) or other basic estimates (e.g. regression coefficient) AND variation (e.g. standard deviation) or associated estimates of uncertainty (e.g. confidence intervals)
☐	☒	For null hypothesis testing, the test statistic (e.g. *F*, *t*, *r*) with confidence intervals, effect sizes, degrees of freedom and *P* value noted<br>*Give P values as exact values whenever suitable.*
☒	☐	For Bayesian analysis, information on the choice of priors and Markov chain Monte Carlo settings
☒	☐	For hierarchical and complex designs, identification of the appropriate level for tests and full reporting of outcomes
☒	☐	Estimates of effect sizes (e.g. Cohen's *d*, Pearson's *r*), indicating how they were calculated

Our web collection on statistics for biologists contains articles on many of the points above.

Software and code

Policy information about availability of computer code

Data collection	No software or code was used to collect data
Data analysis	Raw RNAseq data was aligned using STAR Version 2.5.3a via a DKFZ internal NGS-data processing pipeline. StrataQuest software version 7.0 (Tissue Gnostics) was used for image processing and to analyze whole tissue sections by IF. Single cell RNAseq reanalysis was performed using the scanpy python package version 1.9.3 (https://scanpy.readthedocs.io/en/stable/api.html). Statistical analysis and figure generation was performed using the R statistical programming language (R version 4.1.2) and GraphPad Prism version 9.5 (CA, USA). All R packages used in this study are open source and listed in the Methods section. All code used for analysis is available from the authors on request.

For manuscripts utilizing custom algorithms or software that are central to the research but not yet described in published literature, software must be made available to editors and reviewers. We strongly encourage code deposition in a community repository (e.g. GitHub). See the Nature Portfolio guidelines for submitting code & software for further information.

Data

Policy information about availability of data

All manuscripts must include a data availability statement. This statement should provide the following information, where applicable:

- Accession codes, unique identifiers, or web links for publicly available datasets
- A description of any restrictions on data availability
- For clinical datasets or third party data, please ensure that the statement adheres to our policy

All data relevant to this study is available from the corresponding authors upon request. All processed data including normalized expression data for patient and patient-derived organoids, multiplexed immunofluorescence and experimental results are provided in the Supplementary tables. RNAseq data are available at the European Genome-Phenome Archive https://ega-archive.org/studies/EGAS00001007143. RNAseq data published in Hwang et. al. (17), and Brunton et. al. (31), were obtained from GEO Accession No.: GSE202051 and BioProject Accession No.: PRJNA630992, respectively. Source data for Figures 1-8 and Extended Data Figures 1-10 have been provided as Source Data files.

Human research participants

Policy information about studies involving human research participants and Sex and Gender in Research.

Reporting on sex and gender	Research findings do not apply to one sex or gender only. The gender of each patient was collected by consent and self-reported. Information on gender is provided in Supplementary Tables S1, S2, S4 and S5. For tissue-based findings 171 unique patients were included in the study with 100 males and 71 females taking part. For RNAseq 56 males and 41 females were included in the analysis (Supplementary Table S1 and S4). For multiplexed IF (Supplementary Table S1 and S5) 71 males and 51 females were included in the analysis. For patient-derived organoids, organoids derived from 11 male and 20 female were included in the analysis (Supplementary S10). No gender-based analyses are shown, no significant associations with gender were observed in the data. All source data comprise a patient identifier which can be used to disaggregate the data based on gender.
Population characteristics	Patients with histologically verified Pancreatic Ductal Adenocarcinoma, without distant metastases, who underwent a surgical resection from 2012 to 2020 with stored blood, and cryopreserved and/or FFPE sections, with complete clinical and pathological data, and follow up of at least 24 months. The patients were all operated upon in the Department of General Surgery at the University Hospital of Heidelberg, Germany. Patients initially were staged either as CT UNRESECTABLE disease and had neoadjuvant chemotherapy then resection (including arterial and venous resection); or CT RESECTABLE disease (including venous resection) and had adjuvant chemotherapy. Patients who died within 3 months were excluded. Patients who had any chemoradiation at any time were also excluded. Patient characteristics are summarized in Supplementary Tables S1, S2 , S4 and S5.
Recruitment	Patients were selected according to the following criteria: (i) patients with locally advanced borderline unresectable PDAC (without metastases) who received surgical resection after mFOLFIRINOX or gemcitabine-based therapy without any chemoradiation neoadjuvant therapy; and (ii) patients with resectable PDAC at presentation who had up-front surgical resection without prior chemotherapy and/or chemoradiation (adjuvant chemo-naïve). Post resection patients could receive adjuvant chemotherapy but those who had chemoradiation were again excluded. Consecutive pseudo-anonymised patients with prior consent, and with usable samples of sufficient quality for investigation were identified based on the eligibility criteria. Additional pseudo-anonymised IDs were then ascribed to each patient for experimental investigation, so blinded to all of the investigators and investigations. Clinical correlates were only ascribed once the experimental procedure results were obtained.
Ethics oversight	The study has been approved by the Ethics Committee of Heidelberg University for use of pancreatic cancer tissue (Project Nos. S-018/2020, S-708/2019 and S-083/2021).

Note that full information on the approval of the study protocol must also be provided in the manuscript.

Field-specific reporting

Please select the one below that is the best fit for your research. If you are not sure, read the appropriate sections before making your selection.

☒ Life sciences ☐ Behavioural & social sciences ☐ Ecological, evolutionary & environmental sciences

For a reference copy of the document with all sections, see nature.com/documents/nr-reporting-summary-flat.pdf

Life sciences study design

All studies must disclose on these points even when the disclosure is negative.

Sample size	Sample sizes represent available resected patient material. Statistical tests used in this study are suitable for the number of samples representing each patient group. Sample sizes are provided in the Supplementary tables in Figure Legends.

Data exclusions	Data was not excluded from the study.
Replication	The data included in the study is representative of resected chemo-naive and post-chemotherapy patient samples. All antibodies used in this study have been validated and inter-sample variability has been controlled where possible using internal controls. Multiple tumor sections representing the sample patient sample have been analyzed to replicate findings. Analysis of patient-derived organoids are representative of at least 3 independent experiments.<br><br>All experiments are representative of at least 3 independent biological experiments. H&E and IF images for patient samples or PDOs are representative of at least 3 independent IF experiments on the same region of interest or PDO. The finalized figures represent at least 2 successful replicated experiments.
Randomization	Patients were allocated into groups based on defined clinical criteria.
Blinding	Consecutive pseudo-anonymised patients with prior consent, and with usable samples of sufficient quality for investigation were identified based on the eligibility criteria, defined under "Patients Characteristics" in the Methods. Additional pseudo-anonymised IDs were then ascribed to each patient for experimental investigation, so blinded to all of the investigators and investigations. Clinical correlates were only ascribed once the experimental procedure results were obtained. Subsequent data collection and analysis was not performed blind to the conditions of the experiment.

Reporting for specific materials, systems and methods

We require information from authors about some types of materials, experimental systems and methods used in many studies. Here, indicate whether each material, system or method listed is relevant to your study. If you are not sure if a list item applies to your research, read the appropriate section before selecting a response.

Materials & experimental systems

n/a	Involved in the study
☐	☒ Antibodies
☒	☐ Eukaryotic cell lines
☒	☐ Palaeontology and archaeology
☒	☐ Animals and other organisms
☒	☐ Clinical data
☒	☐ Dual use research of concern

Methods

n/a	Involved in the study
☒	☐ ChIP-seq
☒	☐ Flow cytometry
☒	☐ MRI-based neuroimaging

Antibodies

Antibodies used	CYP3A5 Company: Abcam; Catalogue No.:ab108624; Clone No: EPR4396; Dilution: 1/200; Source: Rabbit<br><br>GATA6 Company: R&D System; Catalogue No.:AF1700 ; Clone No: NA; Dilution: 1/100; Source: Goat<br><br>hENT1 Company: Creative Biolabs; Catalogue No.:CBMAB-E2320-FY; Clone No: CBFYE-1652; Dilution: 1/100; Source: Mouse<br><br>HNF1A Company: Santa Cruz Biotechnology; Catalogue No.:sc-393925; Clone No: F-7; Dilution: 1/100; Source: Mouse<br><br>KRT17 Company: Santa Cruz Biotechnology; Catalogue No.: Sc-393002; Clone No: E-4; Dilution: 1/100; Source: Mouse<br><br>KRT19 Company: Abcam; Catalogue No.: ab7755; Clone No: BA-17; Dilution: 1/400; Source: Mouse<br><br>KRT19 Company: Abcam; Catalogue No.: ab52625; Clone No: EP1580Y; Dilution: 1/400; Source: Rabbit<br><br>KRT5 Company: Thermo Fisher Scientific; Catalogue No.: MA5-12596; Clone No: XM26; Dilution: 1/200; Source: Mouse<br><br>KRT81 Company: Santa Cruz Biotechnology; Catalogue No.: sc-100929; Clone No: 36-Z; Dilution: 1/50; Source: Mouse<br><br>S100A2 Company: Abcam; Catalogue No.: Ab109494; Clone No: EPR5392; Dilution: 1/200; Source: Rabbit
Validation	Antibodies were validated by manufacturer and selected peer reviewed publications. Internal controls were used to validate the positivity of the results.<br><br>CYP3A5 Company: Abcam; Catalogue No.:ab108624; Clone No: EPR4396; Dilution: 1/200; Source: Rabbit; Website: https://www.abcam.com/products/primary-antibodies/cyp3a5-antibody-epr4396-ab108624.pdf; Citation: Noll EM et al. Nat Med. 2016 Mar;22(3):278-87. doi: 10.1038/nm.4038. Epub 2016 Feb 8. PMID: 26855150; PMCID: PMC4780258.<br><br>GATA6 Company: R&D System; Catalogue No.:AF1700 ; Clone No: NA; Dilution: 1/100; Source: Goat; Website: https://www.rndsystems.com/products/human-gata-6-antibody_af1700?gclid=Cj0KCQjw1rqkBhCTARIsAAHz7K2eqwoZ8c3IUo5rtyk0-VMKnmIWIj36Ki7ZFPuK04j4L5_QX_nRlAsaAiuDEALw_wcB&gclsrc=aw.ds#product-datasheets; Citation: Lee H et al. EBioMedicine. 2021 Mar;65:103218. doi: 10.1016/j.ebiom.2021.103218. Epub 2021 Feb 25. PMID: 33639403; PMCID: PMC7921470.

hENT1 Company: Creative Biolabs; Catalogue No.:CBMAB-E2320-FY; Clone No: CBFYE-1652; Dilution: 1/100; Source: Mouse; Website: https://www.antibody-creativebiolabs.com/anti-slc29a1-monoclonal-antibody-cbfye-1652-45515.htm; Citation: NA

HNF1A Company: Santa Cruz Biotechnology; Catalogue No.:sc-393925; Clone No: F-7; Dilution: 1/100; Source: Mouse; Website: https://datasheets.scbt.com/sc-393925.pdf; Citation: Taniguchi H et al Oncotarget. 2018 May 25;9(40):26144-26156. doi: 10.18632/oncotarget.25456. PMID: 29899848; PMCID: PMC5995239.

KRT17 Company: Santa Cruz Biotechnology; Catalogue No.: Sc-393002; Clone No: E-4; Dilution: 1/100; Source: Mouse; Website: https://datasheets.scbt.com/sc-393002.pdf; Citation: Kathiriya JJ et al Nat Cell Biol. 2022 Jan;24(1):10-23. doi: 10.1038/s41556-021-00809-4. Epub 2021 Dec 30. PMID: 34969962; PMCID: PMC8761168.

KRT19 Company: Abcam; Catalogue No.: ab7755; Clone No: BA-17; Dilution: 1/400; Source: Mouse; Website: https://www.abcam.com/products/primary-antibodies/cytokeratin-19-antibody-ba-17-ab7755.pdf; Citation: Husanie H et al. Cell Death Dis. 2022 Dec 27;13(12):1074. doi: 10.1038/s41419-022-05519-9. PMID: 36572673; PMCID: PMC9792466.

KRT19 Company: Abcam; Catalogue No.: ab52625; Clone No: EP1580Y; Dilution: 1/400; Source: Rabbit; Website: https://www.abcam.com/products/primary-antibodies/cytokeratin-19-antibody-ep1580y-cytoskeleton-marker-ab52625.pdf; Citation: Aktas RG et al Cells. 2022 Nov 27;11(23):3797. doi: 10.3390/cells11233797. PMID: 36497057; PMCID: PMC9741396.

KRT5 Company: Thermo Fisher Scientific; Catalogue No.: MA5-12596; Clone No: XM26; Dilution: 1/200; Source: Mouse; Website: https://www.thermofisher.com/order/genome-database/dataSheetPdf?producttype=antibody&productsubtype=antibody_primary&productId=MA5-12596&version=322; Citation: Hao S, et al mBio. 2020 Nov 6;11(6):e02852-20. doi: 10.1128/mBio.02852-20. PMID: 33158999; PMCID: PMC7649230.

KRT81 Company: Santa Cruz Biotechnology; Catalogue No.: sc-100929; Clone No: 36-Z; Dilution: 1/50; Source: Mouse; Website: https://datasheets.scbt.com/sc-100929.pdf; Citation: Muckenhuber A, et al. Clin Cancer Res. 2018 Jan 15;24(2):351-359. doi: 10.1158/1078-0432.CCR-17-2180. Epub 2017 Nov 3. PMID: 29101303.

S100A2 Company: Abcam; Catalogue No.: Ab109494; Clone No: EPR5392; Dilution: 1/200; Source: Rabbit; Website: https://www.abcam.com/products/primary-antibodies/s100-alpha-2s100a2-antibody-epr5392-ab109494.pdf; Citation: Chen Y, et al Front Immunol. 2021 Nov 23;12:758004. doi: 10.3389/fimmu.2021.758004. PMID: 34887861; PMCID: PMC8650155.
