# Supplementary figures and images for: Persister cell phenotypes contribute to poor patient outcomes after neoadjuvant chemotherapy in PDAC
Source: Nat Cancer. 2023 Sep 7;4(9):1362–81. doi: 10.1038/s43018-023-00628-6 (PMC10518256; doi:10.1038/s43018-023-00628-6)

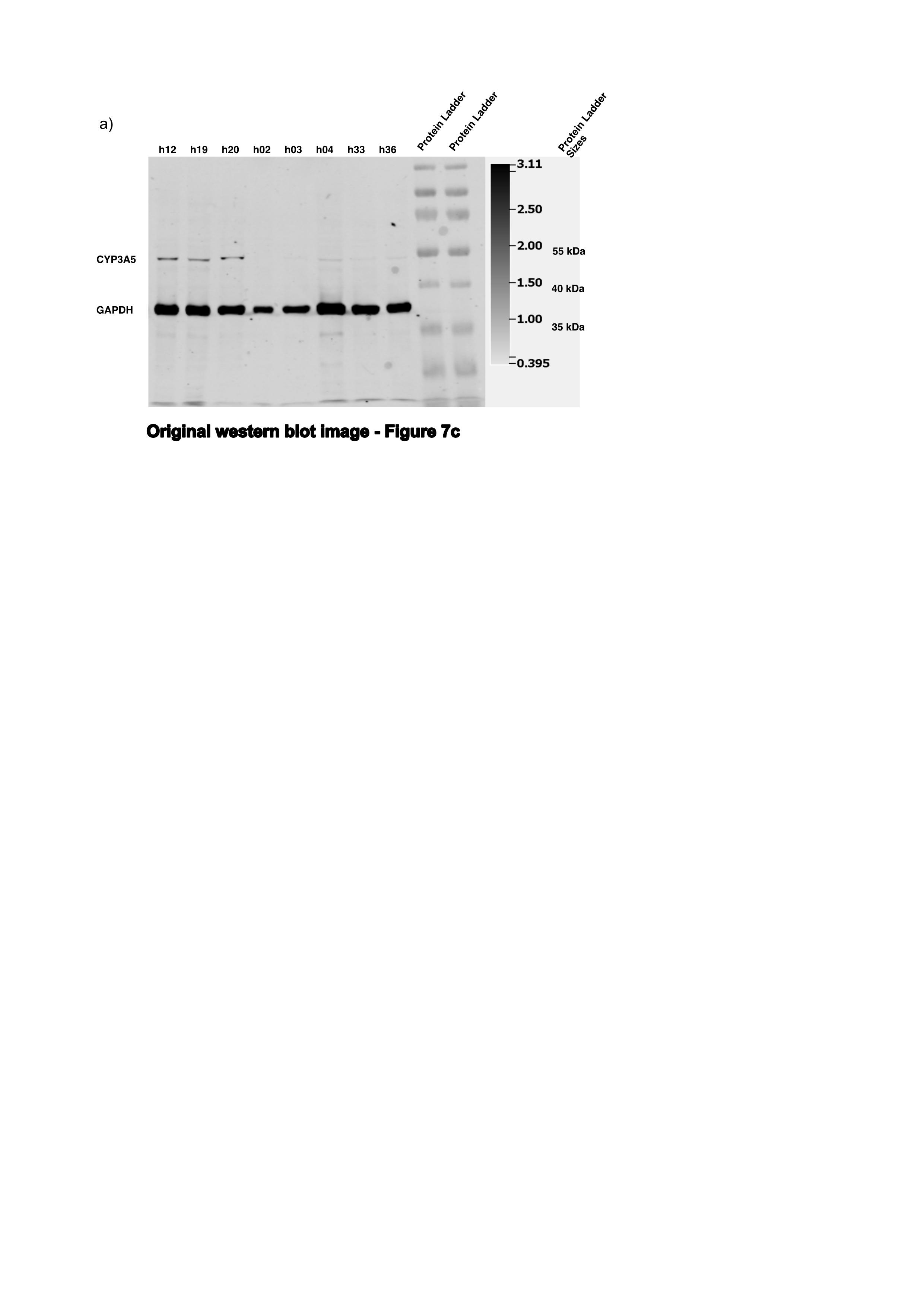

Supplement: Supplementary file 20 — Uncropped blot [file 43018_2023_628_MOESM20_ESM.jpg]
